# Microfluidic Chip‐Assisted Upconversion Luminescence Biosensing Platform for Point‐of‐Care Virus Diagnostics

**DOI:** 10.1002/adhm.202303897

**Published:** 2024-03-15

**Authors:** Yuan Liu, Xinyue Lao, Man‐Chung Wong, Menglin Song, Huang Lai, Pui Wang, Yingjin Ma, Lihua Li, Mo Yang, Honglin Chen, Jianhua Hao

**Affiliations:** ^1^ Department of Applied Physics The Hong Kong Polytechnic University Kowloon Hong Kong 999077 China; ^2^ Department of Biomedical Engineering The Hong Kong Polytechnic University Kowloon Hong Kong 999077 China; ^3^ State Key Laboratory for Emerging Infectious Diseases Department of Microbiology LKS Faculty of Medicine The University of Hong Kong Pokfulam Hong Kong 999077 China; ^4^ Research Centre for Nanoscience and Nanotechnology The Hong Kong Polytechnic University Kowloon Hong Kong 999077 China

**Keywords:** microfluidic chip‐assisted, point‐of‐care, upconversion luminescence, virus diagnostics

## Abstract

Epidemics caused by multiple viruses continue to emerge, which have brought a terrible impact on human society. Identification of viral infections with high sensitivity and portability is of significant importance for the screening and management of diseases caused by viruses. Herein, a microfluidic chip (MFC)‐assisted upconversion luminescence biosensing platform is designed and fabricated for point‐of‐care virus detection. Upconversion nanoparticles with excellent stability are successfully synthesized as luminescent agents for optical signal generation in the portable virus diagnostic platform. The relevant investigation results illustrate that the MFC‐assisted virus diagnostic platform possesses outstanding performance such as good integration, high sensitivity (1.12 pg mL^−1^), ease of use, and portability. In addition, clinical sample test result verifies its more prominent virus diagnostic properties than commercially available rapid test strips. All of these thrilling capabilities imply that the designed portable virus diagnostic platform has great potential for future virus detection applications.

## Graphical abstract

A microfluidic chip (MFC)‐assisted upconversion luminescence biosensing platform is designed and fabricated for point‐of‐care virus detection. Relevant investigation results illustrate that the MFC‐assisted virus diagnostic platform possesses outstanding performance, such as good integration, high sensitivity, ease of use, and portability, implying great potential for future portable virus detection applications.

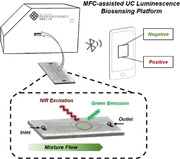

## 1 Introduction

Throughout history, human health and medical systems have repeatedly faced significant threats from various viruses. Among them, some widespread viruses have brought painful recollections for all mankind. Smallpox was a virulent infectious disease caused by the smallpox virus and resulted in severe toxemia and hundreds of millions of deaths centuries ago.^[^ ^1^ ^]^ The Spanish flu pandemic was mainly caused by a flu virus similar to influenza A virus subtype H1N1 (A/H1N1) and gave rise to tremendous distress to the people of Europe.^[^ ^2^ ^]^ In recent years, some known viruses, such as the Ebola virus, Zika virus, severe acute respiratory syndrome coronavirus 2 (SARS‑CoV‑2), and the Mpox virus, have generated enormous influence on the society of human beings.^[^ ^3^, ^4^, ^5^, ^6^, ^7^ ^]^ Therefore, the detection of viruses possesses immense significance in controlling virus transmission ahead of time.

Conventional methods involving polymerase chain reaction (PCR) and lateral flow assays (LFA) are widely implemented for virus detection. By means of amplification for certain gene segments, PCR has an ultrahigh sensitivity of around 100 copies per milliliter, which is a gold‐standard method for virus diagnostics. However, PCR has drawbacks of being time‐consuming for several hours, high detection cost, need for professional operators, and risk of unexpected virus diffusion.^[^ ^8^, ^9^, ^10^ ^]^ Through the development of PCR diagnosis, Chang et al. utilized a non‐optical multiplexed PCR method to detect the dengue virus with a fairly high detection sensitivity. However, the assay time still reaches about 90 min, which limits the application of rapid diagnosis.^[^ ^11^ ^]^ LFA is a relatively rapid detection method using the colloidal gold lateral flow bonding to the modified nitrocellulose membrane.^[^ ^12^ ^]^ Nonetheless, the diagnosis sensitivity of LFA is quite low, which poses disadvantages in terms of early and accurate virus detection. Jin et al. reported the developed upconversion nanoparticles (UCNPs)‐based LFA strips for specific virus and protein detection. However, the diagnosis sensitivity and convenience of this device also need to be improved before it can be widely used.^[^ ^13^, ^14^ ^]^

To address the existing limitations of traditional diagnostic methods, numerous innovative biosensors have been developed for virus detection. Chailapakul et al. employed a paper‐based fluorescent sensor for hepatitis C virus detection, however, the luminous stability of the used organic fluorochrome could not keep relatively high performance in some complex environments.^[^ ^15^ ^]^ By utilizing the effect of fluorescence resonance energy transfer (ET), our group proposes and develops some novel strategies for highly sensitive virus detection. However, the operation procedures required professional apparatus and personnel, which also limited the application to some extent.^[^ ^16^, ^17^, ^18^ ^]^ Therefore, achieving the virus diagnosis involving stabilization, sensitivity, and convenience is of significance for the application in virus detection.

The microfluidic chip (MFC) is a manufactured device consisting of microchannels that are patterned in a specific manner. These microchannels enable the controlled flow of fluids, allowing them to pass through different channels and establish connections with the external environment at designated inlets and outlets. This lab‐on‐a‐chip MFC device possesses numerous features involving programmed functionality, diminutive and exquisite, and biological relevance, which is beneficial for the improvement of integration and portability.^[^ ^19^, ^20^, ^21^, ^22^ ^]^ Furthermore, the MFC with separation functions has been widely used for biosensing areas involving particle filtration and cell sorting.^[^ ^23^, ^24^ ^]^ Hence, by utilizing MFC, it is possible to isolate all targeting biomarkers present in a given sample, leading to a substantial enhancement in detection sensitivity without the requirement of amplification techniques. Sandwich structure is broadly employed for the capture of targeting biomarkers in optical immunoassays, in which a luminescent agent is involved for optical sensing.^[^ ^25^, ^26^, ^27^, ^28^, ^29^ ^]^ Owing to the transition of 4f electrons in the f‐f or between the f‐d configuration of lanthanide luminescent ions, UCNPs doped with lanthanide ions illustrate fairly unaffected and steady luminescent performance.^[^ ^30^, ^31^, ^32^, ^33^, ^34^ ^]^ The mature research methods for the modification of UCNPs have vastly expanded their application in the field of biosensing.^[^ ^35^, ^36^, ^37^ ^]^

In addition, point‐of‐care detection is a near‐patient biosensing technology that possesses numerous merits and has attracted widespread research interest.^[^ ^38^ ^]^ For instance, researchers from Harvard Medical School propose a luminescence compact in vitro diagnostics for virus detection, but the sample treatment of purification and separation is still relatively inconvenient, which restricts the practical application for virus diagnosis.^[^ ^39^ ^]^ Therefore, it is imperative to balance these advantages of biosensing technologies and achieve rapid, sensitive, and portable virus diagnosis.

Herein, we propose a novel strategy for an MFC‐assisted point‐of‐care platform for portable virus diagnosis. The synthesized and modified UCNPs possess good performance of luminescence and steady fluorescence. The choreographed MFC was triumphantly designed and fabricated with superior properties, which accelerated the sensitivity and convenience of virus detection. Compared with the detection approach in liquid form specimens in cuvette, the designed MFC concentrates the test samples into the solid form, which enhances the luminescent intensity of conjugated UCNPs and improves the detection sensitivity to a large extent. Taking the nucleocapsid protein (N protein) of SARS‑CoV‑2 as the diagnostic object, the detection performance of the fabricated MFC‐assisted portable virus detection platform was evaluated and compared with traditional LFA. The result indicated that the MFC‐assisted portable virus detection platform possessed relatively superior sensitivity (≈1.12 pg mL^−1^), good integration, and portability. Moreover, related clinical N protein samples from medical laboratories were employed for assessing the practical virus diagnostic performance. These various excellent performances implied that the proposed MFC‐assisted portable virus diagnostic platform possessed broad prospects for practical application in virus detection.

## 2 Results and Discussion

### 2.1 Mechanism of MFC‐Assisted Diagnostic Platform

**Figure 1:**
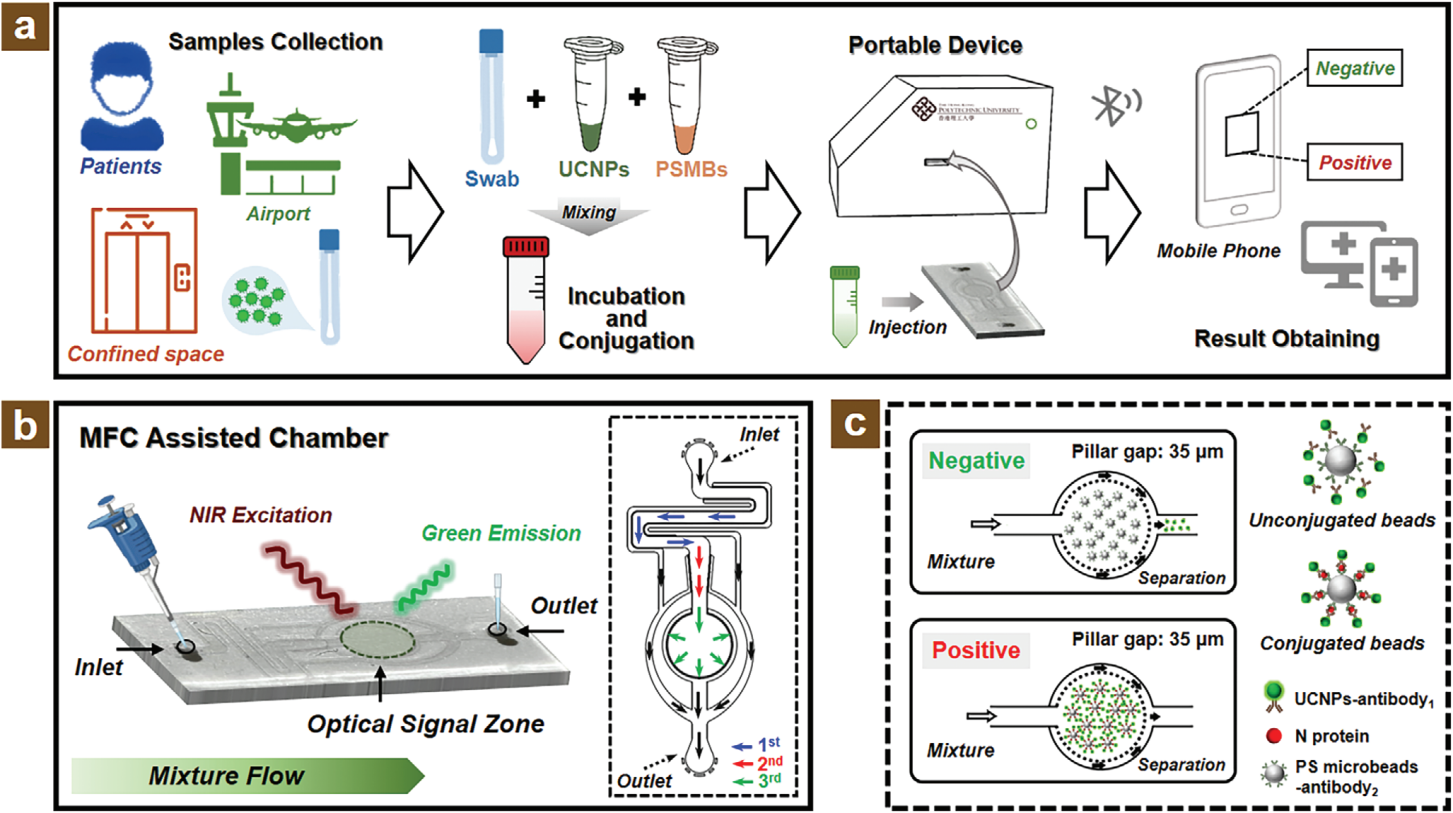
Schematic diagram for MFC‐assisted diagnostic platform. a) Illustration of virus detection procedures including swabs collection, mixing, and incubation for pre‐prepared reagents, samples injection, and test result acquiring. b) The mixture flow and optical signal generation for the MFC‐assisted chamber. c) The fundamental principle of MFC with separation and concentrating function for virus detection.

For the purpose of point‐of‐care and portable virus detection, the MFC‐assisted diagnostic platform was designed and utilized for the detection of N protein of SARS‐CoV‐2 as shown in **Figure** **1**. Specifically, it is illustrated in Figure 1a that the viral samples were collected from the patients, the airport, and some confined spaces such as elevators or washrooms. Then the obtained swabs with or without N protein were mixed with the prepared diagnostic chemicals including modified UCNPs and polystyrene (PS) microbeads (abbreviating to PSMBs in Figure 1a). The mixture was incubated for several minutes and subsequently formed a sandwich immunoassay consisting of UCNPs, N protein, and PS microbeads. Next, the designed MFC‐assisted chamber injected with an incubated mixture will be inserted into a portable detection device for virus diagnosis. Ultimately, the test result can be acquired from a mobile phone via Bluetooth signal transmission and the entire procedure can be achieved within 15 min. In detail, the schematic diagrams and mechanism of MFC assisted chamber are exhibited in Figure 1b,c.

As shown in Figure 1b, the incubated mixture was injected into the inlet of the chip via a convenient pipette and redundancy outflow from the outlet. From the optical signal zone, the green emission signals were observed under the 980 nm near‐infrared (NIR) incident excitation while the existence of N protein. The right‐side insert illustrates a choreographed three‐stage separation system for the unconjugated UCNPs filtration and bounded sandwich PS microbeads concentrating. Moreover, Figure 1c illustrates the separation and concentrating mechanism of the MFC‐assisted chamber. The negative result means that the unconjugated beads stay in the filtration zone with no photoluminescence (PL) signal and the positive result means that the conjugated beads exhibit the PL signals under NIR laser excitation. To sum up, it can be illustrated that these thrilling properties make it a potential platform for portable virus diagnostics.

### 2.2 Morphology and Modification Characterizations

**Figure 2:**
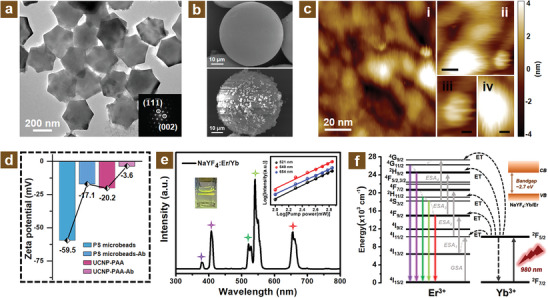
The characterizations of micro‐scale morphology, modification property, and upconversion photoluminescence performance. a) TEM image of NaYF_4_:Er^3+^/Yb^3+^ UCNPs. Insert showing the FFT image under the high‐resolution TEM mode. b) SEM images of PS microbeads with (bottom) and without (top) N protein. c) AFM images of the conjugated antigen and antibody for N protein. d) Zeta potential of PS microbeads and UCNPs before and after conjugation with the antibody. e) NaYF_4_:Er^3+^/Yb^3+^ UCNPs PL spectrum ranges from UV (300 nm) to NIR (800 nm). Left inset: the optical photo of UCNPs (≈1 mg mL^−1^) dispersed in cyclohexane under 980 nm excitation. Right inset: the power‐dependent emission spectra illustrating the two‐photon upconversion process. f) Probable upconversion energy transfer routes of co‐doped Er^3+^ and Yb^3+^ ions.

In order to investigate the morphology of diagnostic probes, various characterization techniques were carried out including transmission electron microscope (TEM), scanning electron microscope (SEM), atomic force microscope (AFM), and zeta potential measurement. From **Figures** **2**a and S1, Supporting Information, it can be observed that the NaYF_4_:Er^3+^/Yb^3+^ UCNPs were synthesized with particle size around 300 nm. The corresponding high‐resolution TEM pictures (Figure S2, Supporting Information) and related fast Fourier transform (FFT) images indicated that the synthesized UCNPs possess good crystalline structure. The relevant Miller indexes were calculated and identified as ($\bar{1}$1$\bar{1}$) and (002) as shown in the inset of Figure 2a. In order to investigate the surface modification of synthesized UCNPs, the Fourier transform infrared spectroscopy was implemented as shown in Figures S3 and S4, Supporting Information. It can be observed that the original oleic acid ligand was removed after acid treatment, and the polyacrylic acid was successfully coated on the UCNPs during subsequent modification. The PS microbeads (Figure S5, Supporting Information) exhibited a well‐transparent capability, which is beneficial to optical signal acquisition in the filtration zone of the MFC chamber. Figure 2b and Figure S6, Supporting Information, illustrate the SEM images of PS microbeads with or without N protein conjugation. From the top half image of Figure 2b, it can be observed that a small amount of UCNPs adhere to the surface of the PS microbeads, but the bottom half of SEM images indicate the conjugation situation between the PS microbeads and UCNPs.

On account of the strong destructiveness of the electron beam, the AFM with nondestructive performance for biological samples was utilized for the morphology characterization of the protein form objectives, such as N protein antigen, related antibody, and the conjugation conditions. From Figure 2c, it can be observed that the N protein antigen conjugated well with its related antibody, which was advantageous for the formation of the PS microbeads, N protein antigen, and modified UCNPs sandwich structure. As can be seen from Figure 2d, the measurement of zeta potential further demonstrated the modification property of antibodies onto the surface of PS microbeads and UCNPs. It can be observed that the zeta potential of PS microbeads decreased from −59.5 to −17.1 mV after the modification of the antibody, and the zeta potential of UCNPs was also decreased due to the antibody combination from −20.2 to −3.6 mV. This variation of zeta potential proved that the specific antibodies were conjugated onto the surface of the PS microbeads and UCNPs well. In addition, the dynamic light scattering spectrum as shown in Figure S7, Supporting Information, was also employed to investigate the modification condition for prepared UCNPs‐Ab. The average size for modified UCNPs is determined to be around 327 nm, which is larger than the unmodified UCNPs and further verifies the good modification of antibodies with UCNPs.

### 2.3 PL Performance of Synthesized UCNPs

UCNPs are used as photovisualization reagents for N protein detection due to their less background interference in NIR compared to UV excitation. The related photoluminescence properties were investigated as shown in Figure 2e. Under NIR (980 nm) light excitation, the emissions of Er/Yb co‐doped UCNPs exhibited five peaks located at 378, 408, 521, 540, and 654 nm, which were originated from the excited state of ^4^G_11/2_, ^2^H_9/2_, ^2^H_11/2_, ^4^S_3/2_, and ^4^F_9/2_ to the ground state of ^4^I_15/2_, respectively. The strongest emission peak was located at 540 nm indicating the emission color of the UCNPs (left inset of Figure 2e) utilized for the posterior virus diagnosis. The power‐dependent emission spectra were investigated for the participation of upconversion photons. As shown in the inset of Figure 2e, the slopes of fitted curves are 1.98, 1.93, and 1.84 for the emission peaks of 521, 540, and 654 nm, respectively. According to a relationship of *I ∝ P^n^*, *I* stands for the luminescence intensity, *P* represents the pump power, and *n* is the average participant upconversion photons.^[^ ^40^, ^41^, ^42^ ^]^ After the logarithmic treatment of pump power and photoluminescence intensity, the data points of logarithmic *I* and *P* illustrated linear response, and the slopes were related to the photons. As a result, it can be indicated that a two‐photon upconversion process takes place in the NaYF_4_:Er^3+^/Yb^3+^ UCNPs.

The upconversion energy transfer process of codoped Er^3+^/Yb^3+^ ions is exhibited in Figure 2f. It mainly consisted of several processes, such as ET, ground state absorption (GSA), and excited state absorption (ESA), for the entire upconversion transitions. The upconversion mechanism of red emission located at 654 nm is mainly: Ground state level ^4^I_15/2_ jumps to ^4^I_11/2_ level after absorbing 980 nm excitation light. Next, the excited state level ^4^I_11/2_ relaxes non‐radioactively to the ^4^I_13/2_ and then jumps to ^4^F_9/2_ because of the ESA process. Finally, the radiative relaxation from the ^4^F_9/2_ level to the ground state brings the red light (654 nm) emission. In terms of the two green emissions, under a 980 nm laser excitation, the population of Er^3+^ ions pumps to the excited state of ^4^I_11/2_ from ground state ^4^I_15/2_ and then transitions to ^4^F_7/2_ through the ESA process. It happens the non‐radioactively process from the ^4^F_7/2_ to ^2^H_11/2_ level and ^2^H_11/2_ to ^4^S_3/2_ level. Ultimately, the radiation transitions from the ^2^H_11/2_ and ^4^S_3/2_ levels to the ground state level were observed to be green emissions of 521 and 540 nm, respectively.

In addition, two violet emissions (378 and 408 nm) are probably originated from the excited state of ^4^G_11/2_ and ^2^H_9/2_ transitions to the ^4^I_15/2_ ground state.^[^ ^43^ ^]^ These above emission processes are enhanced due to the doping of the Yb^3+^ ions via the various ET processes. The relevant bandgap of the UCNPs matrix was measured and calculated to around 2.7 eV from the Tauc‐plot as shown in Figure S8, Supporting Information.^[^ ^44^, ^45^ ^]^ Moreover, the lifetimes are shown in Figure S9, Supporting Information, where diverse emission peaks of 378, 408, 521, 540, and 654 nm were also studied through the photoluminescence decay time. It reveals that the lifetime is 180.6, 207.1, 338.3, 321.1, and 570.7 µs, respectively.

### 2.4 MFC with Separation and Concentrating Properties

The fabricated MFC was utilized as a sample chamber in the diagnostic platform, and several MFC channels were designed for separation and concentrating functions. For the MFC‐1, as shown in Figure S10, Supporting Information, the pillar gap of the first‐order filtration system was 25 µm, and the second‐order filtration system possessed a 35 µm pillar gap. However, the drawback was that this layout of MFC usually occurred some background signals as shown in Figures S11 and S12, Supporting Information, which is probably attributed to the fewer stages of the separation system. Therefore, a three‐order filtration system was designed and fabricated as the MFC‐2 as shown in Figure S13, Supporting Information. In this MFC, the pillar gaps of first, second, and third‐order filtration systems were 15, 25, and 35 µm, respectively. This configuration of MFC possessed better primary separation properties compared with the MFC‐1, but the operation time is longer and the cleaning process is inconvenient to a large extent as shown in Figures S14 and S15, Supporting Information.

**Figure 3:**
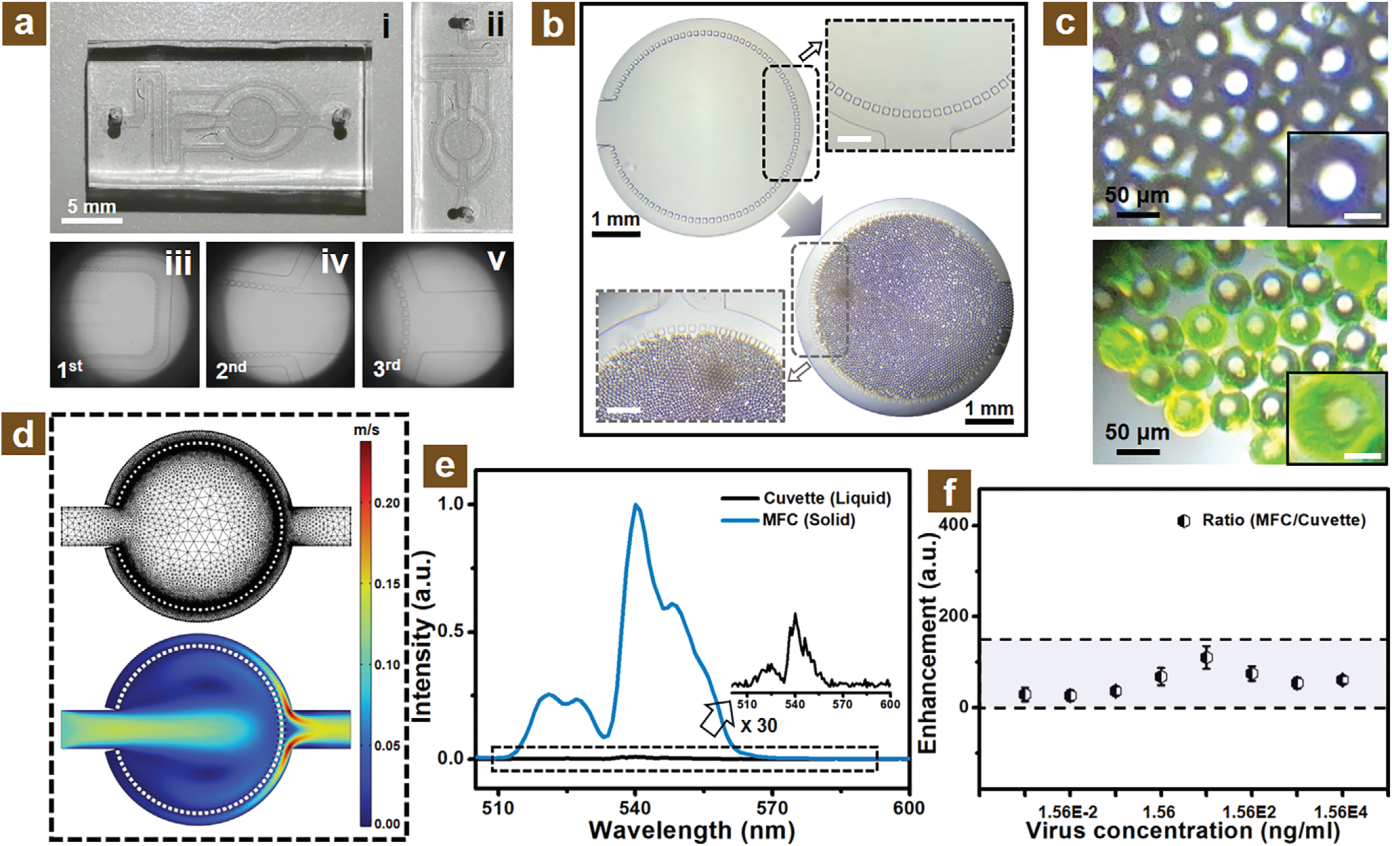
The separation and concentrating performance of designed microfluidic chips. a) The channels of designed microfluidic chips and related microscope photos. b) The separation property of microfluidic chips. c) Optical microscope images of PS microbeads with (bottom) and without (up) N protein conjugation. d) The velocity profile via COMSOL simulation and related mesh calculation. e) The emission spectra of the conjugated UCNPs in cuvette or MFC mode. f) The emission enhancement of MFC mode conjugated UCNPs with different virus concentrations.

In that case, the MFC‐3 with better‐designed channels, as shown in **Figure** **3**a, was selected as the optimized MFC, which the pillar gap of the three‐order filtration system is 15, 25, and 35 µm, respectively, and the AutoCAD design drawing is described in Figure S16, Supporting Information. It can be observed from the bottom panels in Figure 3a that the fabricated PDMS channels connected well with the glass slide due to the chemical bond. The (i) to (iii) images of Figure S16, Supporting Information, indicate the three‐order separation pillars of the designed MFC, which represent the primary and second separation of unconjugated UCNPs, and the conjugated PS microbeads filtration, respectively. In addition, the reasons for the selection of PDMS and glass were high transparency and chemical bonding properties, which were appropriate for the NIR excitation application.

The separation performance of these designed chips is illustrated in Figure 3b and Figure S17a, Supporting Information. It can be obviously seen that the conjugated PS microbeads were filtrated well in the circular zone, which implied the splendid double function, separation, and concentration, possessed great performance for the choreographed MFC. As shown in Figure S17b, Supporting Information, the cleaning process also indicates that this MFC possesses well‐cleanable performance, which implies great potential application for the biosensing field. The related images as shown in Figure S18, Supporting Information, exhibit the concentrating performance of this MFC. It can be obviously observed bright light at the concentrating zone of MFC compared with the dispersion in the liquid condition of conjugated PS microbeads after the NIR irradiation. In addition, as shown in Figure 3c, the optical images from the bright field microscope further illustrated that the PS microbeads illuminated bright green emission when conjugated with UCNPs via N protein, which was also evidence for the conjugation and concentrating of connected PS microbeads with UCNPs. Moreover, as shown in Figure 3d and Figure S19, Supporting Information, the color distribution of designed MFC with and without PS microbeads from the COMSOL simulation appeared to be uniform indicating that the velocity gradient was undiversified, which is owing to the circular design of the filtration pillars.

Due to the employing MFC chamber, the related photoluminescence intensity of emission spectra exhibited enhancement performance as shown in Figure 3e. The corresponding normalized spectra indicate that the PL intensity will be improved over 100 times after the concentrating of conjugated UCNPs onto the surface of PS microbeads compared with the cuvette sample, which was calculated from the PL intensity ratio at the emission peak of around 540 nm. Furthermore, with the different concentrations of virus conjugation, all of the PL intensities of the MFC mode demonstrated enhancement performance compared with the cuvette form as shown in Figure 3f and Figure S20, Supporting Information. This PL emission enhancement is originated from the conjugated luminescent PS microbeads concentrating in the separation zone of designed MFC compared with the liquid state of cuvette samples as shown in Figure S21, Supporting Information. This infusive performance of the designed MFC with plentiful merits implied well potential application for the posterior portable virus diagnostic platform.

### 2.5 Diagnostic Performance of MFC‐Assisted Platform

**Figure 4:**
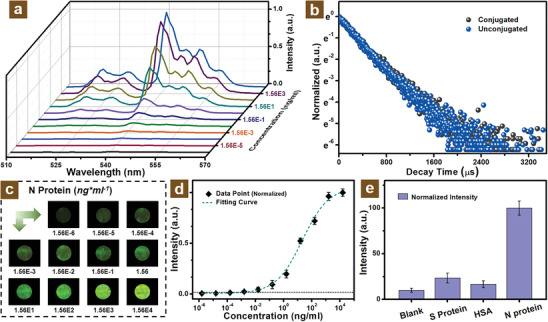
Biosensing performance of diagnostic platform for N protein detection. a) Upconversion emission spectra of concentrated PS microbeads with N protein concentration variation. b) UCNPs decay time with and without N protein conjugation. c) Luminescent photographs of MFC concentrated zone with different concentration N protein conjugation under NIR illumination. d) Detection limit of MFC‐concentrated PS microbeads conjugated with N protein. e) Specificity test N protein detection with PS microbeads and modified UCNPs.

In terms of virus detection for N protein, the investigation of detection limit and conjugation specificity is essential for the evaluation of diagnostic performance.^[^ ^46^, ^47^, ^48^ ^]^ As shown in **Figure** **4**a, as an increase in N protein concentration, the PL intensity showed a growth trend, which was attributed to the raising of the conjugated UCNPs onto the surface of the PS microbeads. Besides, it can be also observed that the increase in PL intensity tended to tardiness at the high protein concentration range. The related lifetime at the emission peak of 540 nm was studied through the decay curve as shown in Figure 4b. It can be observed that the lifetime of Er^3+^ ions was unchanged before and after the addition of N proteins, which indicated that the conjugation of UCNPs onto the surface of PS microbeads will not impact the luminescent lifetime and also implied it did not occur the energy transfer process (Figure S22, Supporting Information) between the UCNPs and PS microbeads. This may be attributed to the characteristic absorbance wavelength of PS microbeads located at around 260 nm, the π–π* electronic transition of the aromatic ring in the styrene monomer, which was much mismatched with the emission peaks of Er^3+^ ions.^[^ ^49^ ^]^ In addition, the relevant optical images of the MFC concentrated zone shown in Figure 4c also illustrate that luminescent intensity tended to rise as the concentration of conjugated virus increased from the range of 1.56 × 10^−6^ to 1.56 × 10^4^ ng mL^−1^.

As shown in Figure 4d, the data points exhibited the comparative PL intensity at an emission peak of 540 nm as a function of UCNP concentration. These values of PL intensity were normalized with the maximum PL intensity set as standard (1.0). It can be obviously seen that the PL intensity increased with the addition of N proteins same as the tendency of the above emission spectra and optical images. At the low N protein concentration range, the increased trend of PL intensity exhibits tardiness, which probably resulted from the inconspicuous PL emissions of conjugated UCNPs onto the surface of PS microbeads. This phenomenon is also exhibited at the high concentration of virus with the further addition of N protein, which can be mainly attributed to the saturation of PL intensity of UCNPs connected onto the surface of PS microbeads. The detection limit is typically evaluated by the mean values of blank samples plus three times of standard deviation.^[^ ^50^ ^]^ After the assessment of the detection limit for 1.12 pg mL^−1^ as shown in Figure 4d, the fabricated platform illustrated remarkable detection sensitivity, which was expected to possess great feasible application for the virus diagnosis. In addition, the investigation of specificity is of great significance, which manifests the recognition capability of virus diagnostic platforms for specific target antigens. As shown in Figure 4e, three different antigens, including spike protein (S protein), human serum albumin (HSA), and N protein, were tested for specificity research. The concentrations of proteins are diluted to around 10 µg mL^−1^ from the standard samples of S protein (1.48 mg mL^−1^), HAS (10 mg mL^−1^), and N protein (1.56 mg mL^−1^). Among them, these error bars manifest the standard deviations of three independent experiments. These results indicated that the N protein target presented higher PL intensity compared with other antigens targets and blank samples, which implied that the designed sandwich immunoassay possessed good virus diagnostic specificity. These investigations of virus detection for N protein performance indicate that this well‐designed virus detection system is a promising potential application for point‐of‐care virus diagnostics.

### 2.6 Detection Performance Comparison with MFC and LFA

**Figure 5:**
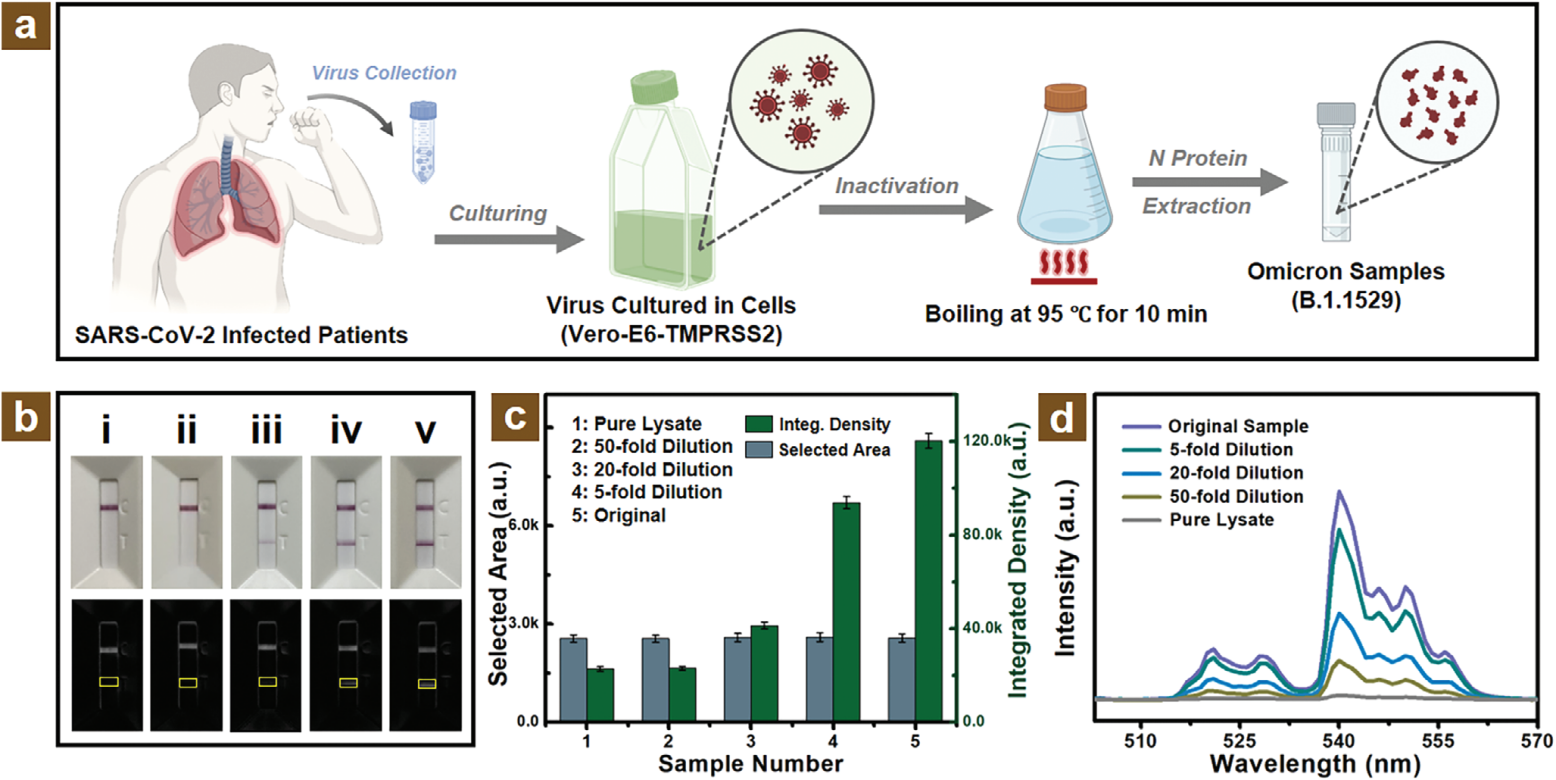
The diagnostic performance comparison of portable MFC assisted platform and LFA rapid test strips for clinical Omicron variant samples. a) The acquiring procedures of clinical viral samples for B.1.1529 Omicron variant N protein. b) The optical photos of LFA rapid test strips with pure lysate (i) and different virus concentration injection (ii–v). Half bottom showing the related converted grayscale images. c) The selected area intensity and the integrated density of the test line for LFA with various sample concentrations and pure lysate via the grayscale images. d) The upconversion emission spectra of the designed MFC diagnostic platform with pure lysate and different concentration clinical samples.

In order to demonstrate the practical detection performance, clinical samples were investigated for our virus diagnostic platform. As shown in **Figure** **5**a, the clinical samples of the Omicron variant were obtained via several procedures from the laboratory of the University of Hong Kong. At first, the isolate of the Omicron (B.1.1.529) variant was acquired from the confirmed patients in Hong Kong. Next, the collected swabs of nasopharyngeal or oropharyngeal specimens were cultured in typical Vero‐E6‐TMPRSS2 cells for replication. The cultured Omicron variant was subsequently boiled at 95 °C for 10 min for virus inactivation. Ultimately, the N protein of the Omicron variant was extracted and acquired for clinical samples. The commercial LFA rapid test strips as shown in Figure 5b were utilized for the N protein detection and verification of the obtained clinical viral samples. With the addition of different concentrations for clinical samples, all of these control lines of LFA strips exhibited a distinct red color indicating that the results of the test line were effective for the N protein detection. For relatively quantitative analysis, the optical images of LFA rapid test strips were converted into grayscale images for investigation.

Figure 5c illustrates the selected area and integrated density of the LFA strips test line of clinical samples with diverse dilution concentrations. It can be seen that the selected area of the test line was basically the same for better comparison. From the original concentration to the 50‐fold dilution concentration, it can be observed the integrated density tended to decrease visibly. However, compared with the 50‐fold dilution sample and the pure lysate, the integrated density values were basically similar, which implied that the detection sensitivity could not reach this dilution viral concentration. Fortunately, the designed MFC‐assisted N protein diagnostic platform manifested excellent performance compared with the commercial LFA rapid test strips. As shown in Figure 5d, the emission intensity also exhibited a downtrend from the original concentration to the dilution clinical sample. However, instead of the inconspicuous distinguishing of 50‐fold dilution sample and pure lysate for commercial LFA rapid test strips, the designed MFC‐assisted virus diagnostic platform possessed significant detection sensitivity, and the emission intensity of 50‐fold dilution clinical sample was distinctly differentiated from the pure lysate compared with commercial LFA strips. This better diagnostic capability may probably attributed to that the targeted antigen conjugated with red color colloidal golds are captured on the test line via lateral flow and observed by the naked eye instead of the UCNPs conjugated PS microbeads concentrating in the central zone and emission with green light under 980 nm laser excitation. Therefore, these perfect diagnostic performances of the MFC‐assisted platform imply it a great potential application for virus detection.

### 2.7 Diagnostic Property for Point‐of‐Care MFC Device

**Figure 6:**
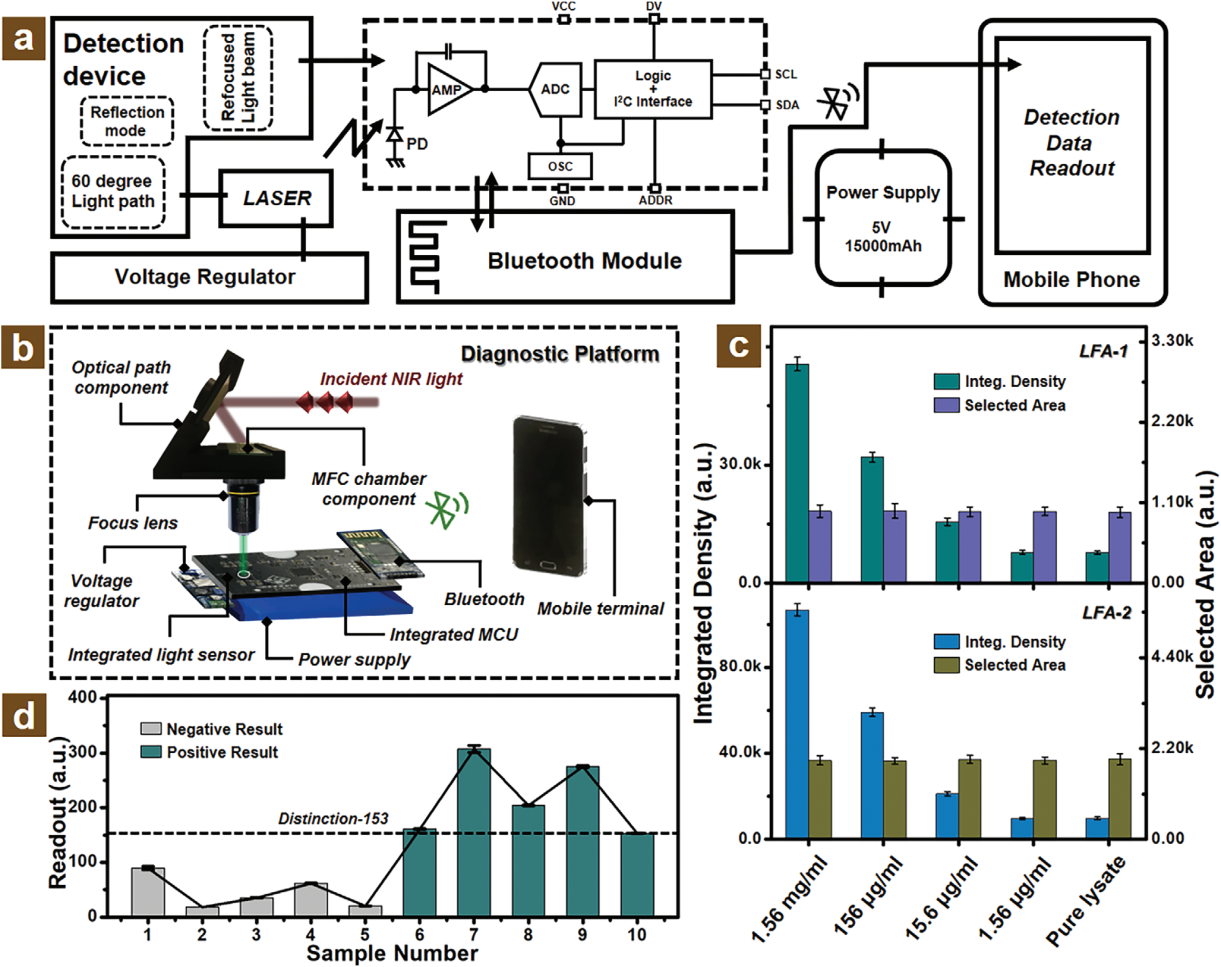
Point‐of‐care MFC‐assisted detection device for N protein diagnosis. a) Flow chart of portable virus diagnostic platform. b) Schematic of diverse components for the elaborate portable device. c) Virus detection property of two different commercial N protein test strips via grey value obtained from selected area. d) Histogram plot of virus sample detection results with negative or positive for the designed portable device.

Furthermore, in order to achieve point‐of‐care virus detection, a portable MFC‐assisted diagnostic platform was designed and fabricated as shown in **Figure** **6**. The flow chart of this designed portable virus detection platform is illustrated in Figure 6a involving different device components. The light sensor is one of the key components of this portable virus diagnostic platform for the signal transformation from optical to electronic as shown in the dashed box via BH‐1750 data sheet. The elaborate MFC was utilized as the sample chamber for the point‐of‐care diagnostic platform as shown in Figure 6b, which consists of NIR incident light from the laser source, optical path component, focus lens, MFC chamber component, voltage regulator, integrated light sensor, power supply, integrated microcontroller unit (abbreviating to MCU in the diagram), Bluetooth module, and mobile terminal. In detail, the assembled laser component generates an NIR incident light and is reflected to the concentrating zone of MFC via a reflecting mirror supported by the 3D printed holder. The separated PS microbeads conjugated with UCNPs were irradiated to a green light beam and focused on the light sensor by the focus lens. The obtained optical signals were transferred to an electric signal and sent to a smartphone through the Bluetooth module. The voltage regulator balanced the voltage of electric components and the power supply with a high capacity of 5 V/15 000 mAh was selected for the longer run time of our portable virus detection platform. All of the management of programs, such as signal transition, data coding, and information sending, were implemented by the integrated microcontroller unit.

Among these components of the designed detection device as shown in the top left corner of Figure 6b and Figure S23, Supporting Information, the angle selection of light path is crucial. It can be obviously observed that the 60‐degree light path was selected as the optimized light path. The reason why chose the reflectance optical mode is that the influence of excitation is minimized compared with the incident optical path mode. The different angles of the light path will also affect the emission light intensity of conjugated UCNPs onto the PS microbeads concentrated in the MCF separation zone. Therefore, three typical angles for convenient fabrication of light paths were investigated as shown in Figure S24, Supporting Information. From Figure S24a, Supporting Information, the schematic diagrams illustrate the three different light path angles including 30, 45, and 60 degrees. In order to study the effect of the different light paths for the monitored emission intensity, the PL emission spectra were studied as shown in Figure S24b, Supporting Information. It can be obviously observed that the emission intensity exhibited an increased trend from the 30 to 60‐degree optical path. For the 60‐degree light path, the emission intensity indicated as the maximum value compared with other reflectance angles, which manifested that the optimized selection of light path was determined to be 60‐degree. In addition, the corresponding properties of integrated light sensors were also studied as shown in Figure S25, Supporting Information. The incident angle will have an effect on the response of light sensors, the best incident angle was determined to be 0 degrees from the related BH‐1750 technical note, which indicated the focused emission light of conjugated UCNPs onto the surface of PS microbeads concentrated in the separation zone of MFC should enshroud the acceptance area of integrated light sensor. As shown in Figure S25a,b, Supporting Information, the detection area of the light sensor was determined to be around 1.5 mm^2^, which was less than the focus emission light (Figure S25c, Supporting Information).

The two commercial LFA rapid test strips were investigated for detection sensitivity via the addition of a standard N protein sample as shown in Figures S26 and S28, Supporting Information. In order to quantitatively analyze the diagnostic performance, these optical photos were converted into the grayscale images shown in Figures S27 and S29, Supporting Information, as well. As shown in Figure 6c, the selected area of the test line of the LFA strip was controlled basically as the same value for better comparison. It can be observed that the integrated density tended to grow with the increased addition of N protein concentration. The tested LFA strips exhibited poor virus detection performance and were estimated at the level of around 1.56 µg mL^−1^, which was similar to commercial LFA strips but well below the sensitivity of our designed MFC‐assisted virus diagnostic platform.^[^ ^51^ ^]^ In addition, ten random N protein samples were also studied on the portable virus diagnostic platform for the negative and positive results acquired from the cell phone for the mobile scene application. As shown in Figure 6d and Figures S30 and S31, Supporting Information, the distinction line between the negative and positive sample was determined to be 153 from the readout of the mobile phone. The two different sample groups of negative or positive indicated significant differences, which possessed well potential application for the diagnostic results.

## 3 Conclusion

In summary, the MFC‐assisted portable N protein diagnostic platform was designed and fabricated successfully. The synthesized NaYF_4_:Er^3+^/Yb^3+^ UCNPs with excellent luminescent stability and uniformity were used as the luminescent indicator for sandwich structure immunoassay. The designed MFC was utilized as the detection chamber of the portable virus diagnostic platform, which illustrated outstanding filtration performance for the conjugated PS microbeads. The fabricated MFC possessed excellent luminescence enhancement properties with a maximum of over 100‐fold than the cuvette samples. Moreover, the cleanable performance of MFC also implied great potential application for the virus diagnosis field. Compared with the typical commercial LFA rapid test strips for virus detection, the MFC‐assisted portable N protein diagnostic platform exhibited a higher biosensing sensitivity of around 1.12 pg mL^−1^. In addition, the clinical samples also manifested that the MFC‐assisted virus detection platform possessed better diagnostic sensitivity compared with the usual commercial LFA rapid test strips. These outstanding detection capabilities implied that the designed MFC‐assisted portable virus diagnostic platform possessed enormous potential applications for the future unpredictable virus detection area.

## Conflict of Interest

The authors declare no conflict of interest.

## Supporting information

Supporting Information

## Data Availability

The data that support the findings of this study are available from the corresponding author upon reasonable request.
