# Supplementary material for: Microfluidic Chip‐Assisted Upconversion Luminescence Biosensing Platform for Point‐of‐Care Virus Diagnostics
Source: Adv Healthc Mater. 2024 Mar 15;13(16):2303897. doi: 10.1002/adhm.202303897 (PMC11468664; doi:10.1002/adhm.202303897)
Supplement: Supplementary file 1 — Supporting Information [file ADHM-13-2303897-s001.pdf]

ADVANCED HEALTHCARE MATERIALS

Supporting Information

for *Adv. Healthcare Mater.*, DOI 10.1002/adhm.202303897

Microfluidic Chip-Assisted Upconversion Luminescence Biosensing Platform for Point-of-Care Virus Diagnostics

*Yuan Liu, Xinyue Lao, Man-Chung Wong, Menglin Song, Huang Lai, Pui Wang, Yingjin Ma, Lihua Li, Mo Yang, Honglin Chen and Jianhua Hao**

Supporting Information

Microfluidic Chip-assisted Upconversion Luminescence Biosensing Platform for Point-of-care Virus Diagnostics

Yuan Liu [a], Xinyue Lao [a], Man-Chung Wong [a], Menglin Song [a], Huang Lai [b], Pui Wang [c], Yingjin Ma [a], Lihua Li [a], Mo Yang [b,d], Honglin Chen [c], Jianhua Hao [a,d*]

[a] *Department of Applied Physics, The Hong Kong Polytechnic University, Kowloon, 999077, Hong Kong, China*

[b] *Department of Biomedical Engineering, The Hong Kong Polytechnic University, Kowloon, 999077, Hong Kong, China*

[c] *State Key Laboratory for Emerging Infectious Diseases, Department of Microbiology, LKS Faculty of Medicine, The University of Hong Kong, Pokfulam, 999077, Hong Kong, China*

[d] *Research Centre for Nanoscience and Nanotechnology, The Hong Kong Polytechnic University, Kowloon, 999077, Hong Kong, China*

**Corresponding author: jh.hao@polyu.edu.hk*

Keywords: upconversion luminescence, microfluidic chip-assisted, point-of-care, virus diagnostics

Experimental Procedures

Chemicals and Materials: Lanthanide acetates including $Y(Ac)_3{\cdot}4H_2O$, $Yb(Ac)_3{\cdot}4H_2O$, $Er(Ac)_3{\cdot}4H_2O$, NH_4F, 1-octacene (ODE), oleic acid (OA), cyclohexane, hydrochloric acid (HCl, 37 %) and Poly(acrylic acid) (PAA, Mw=1800) were purchased from Sigma Aldrich. 1-Ethyl-3-(3-dimethylaminopropyl)-carbodiimide hydrochloride (EDC) and N-hydroxysulfosuccinimide sodium salt (sulfo-NHS) were obtained from TCI. 2-(N-morpholino)ethanesulfonic acid (MES) were purchased from Aladdin. Carboxyl-modified monodisperse polystyrene (PS) microbeads were purchased from Wuxi Rigor Technology CO., LTD. The purified Omicron (B.1.1529) variant of N protein was obtained for the University of Hong Kong, Faculty of Medicine, LKS Center for PanorOmic Sciences. Antibodies and standard N protein of SARS-CoV-2 were purchased from GenScript Biotech Corporation. All the above chemicals were used without further purification. Lateral flow assays rapid test kits-1(LFA-1) were purchased from BioTeke Corporation Wuxi Co., Ltd. And Lateral flow assays rapid test kits-2(LFA-2) were purchased from Hangzhou Sejoy Electronics & Instruments Co., Ltd.

Characterizations: The transmission electron microscopic (TEM) images were obtained by a field emission TEM (JEOL JEM-2100F). The Fourier transform Infrared spectra were acquired from the Bruker Vertex-70 FTIR spectrometer. Scanning electron microscope (SEM) images were recorded on a Tescan MIRA field emission SEM. A scanning probe microscope (Asylum MFP-3D Infinity) was utilized for atomic force microscopy (AFM) measurements. The Zeta potentials and dynamic light scattering spectrum were collected by using a Malvern Zeta Potential Analyzer. Optical photos of PS microbeads were recorded by using Leica DM1750 optical microscopy. The relevant emission and lifetime spectra were measured by EDINBURGH fluorescence spectrometer equipped with a 980 nm pulsed laser from Changchun New Industries Optoelectronics Technology Co., Ltd. The absorbance spectra were collected by using a PERKIN ELMER UV-Vis-NIR spectrometer.

Synthesis of $NaYF_4$:Er^{3+}/Yb^{3+} nanocrystal: The $NaYF_4$:Yb/Er UCNP was synthesized by the coprecipitation method [1,2]. Typically, 1.6 ml of 0.2 M $Y(CH_3COO)_3$, 0.72 ml of 0.2 M $Yb(CH_3COO)_3$, 0.08 ml of 0.2 M $Er(CH_3COO)_3$, 4 ml of OA and 6 ml of ODE were added into a 50-ml flask. The solution was slowly heated from room temperature to 150 °C and maintained for 40 minutes under magnetic stirring. Then the reaction mixture was cooled to room

temperature, followed by injecting a mixture of 1 ml of 1 M NaOH-methanol solution and 3.3 ml of 0.4 M NH_4F-methanol solution. The mixture was then heated to 50 °C for 30 minutes to evaporate methanol. After that, the mixture was heated to 100 °C with a 10-minute vacuum degassing to evaporate the residual moisture. The flask was then heated to 290 °C under the protection of argon gas and maintained for 2 hours before cooling to room temperature. The synthesized UCNPs were precipitate by adding 5ml of ethanol and collected by centrifugation. The UCNPs were further purified with cyclohexane and ethanol three times and dispersed in cyclohexane for further usage.

Surface modification of UCNP: The as-synthesized UCNP was precipitated in 15 mL ethanol with 112 μL HCl (2 M). After 30 min, the UCNPs were precipitated by centrifugation and redispersed in 15 mL ethanol with 11.2 μL HCl (2 M), and sonicated for 30 min. Then the ligand-free UCNP were collected by high-speed centrifugation. After that, PAA (20 mg) dissolved in NaOH (0.2 M) was added to the ligand-free UCNPs. and stirred overnight. UCNP-PAA were collected by high-speed centrifugation and dispersed in water (1 mL) for storage.

Conjugation of UCNP-PAA with antibody: The conjugation of antibody with UCNP-PAA or PS microbeads was carried out by traditional chemical methods [3]. UCNP-PAA (200 μL) was buffer-exchanged in MES buffer (pH 5, 500 μL). After that, EDC (3 mg) and NHS (6 mg) were added to the csUCNPs under stirring for 30 min. Then, 0.1 mg of antibody was added to the tube and stirred for another 2.5 h to ensure a complete coupling reaction. The antibody conjugated UCNP was collected by high-speed centrifugation and washed with water three times.

Conjugation of PS microbeads with antibody: First, 1mg of carboxy group modified PS microbeads were distilled in 1 mL MES. 10 μL of 10 mg/mL EDC/NHS was added and the solution was incubated at 37 °C for 30 min. Then the PS microbeads were precipitate and redispersed in PBS, with the addition of 0.3 mg Antibody in 800 μL PBS. The mixture was incubated at 37 °C for 1h. After the reaction, 5mg/mL of BSA was added and incubated for another 1 hour. The resultant antibody conjugated PS microbeads were washed and resuspended in PBS for further usage.

Microfluidic chips fabrication: The microfluidic chips were fabricated by the conventional soft lithography and reverse molding procedures [4–6]. In details, the manufacture processes were illustrated as follows: At first, the wafer mold was fabricated by the photolithography method.

The designed pattern was drawn through the AutoCAD software and then manufactured to film Mask. For the specific wafer mold photolithography process, the 4-inch wafer was cleaned via acetone, IPA, DI Water, and N_2 gas. The SU-8 photoresist around 4 ml was next spun coating onto the surface of the wafer with no bubble. Then the photoresist coated wafer was treated for soft bake under 65 & 95 degrees Celsius and follow by pattern transfer via the photoetching machine. Subsequently, the post bake was carried out with 65 & 95 degrees Celsius and the baked wafer was executed for developing with SU-8 developer, IPA, DI Water, and N_2 gas. After the final hard bake for 150 degrees Celsius, the wafer was cooling down to room temperature for the later processes. The PDMS-glass MFC was fabricated via the followed steps: the obtained wafer mold under lithography was evaporated with a thin organic film for better demolding, and follow by the PDMS pouring and thermocuring with the ratio of 1:10 for the curing agent and base. Next, the cured PDMS with designed pattern was implemented with demolding and clipping for the following plasma treatment. And the plasma treated PDMS chips and glasses were bonded and heated for 85 degrees Celsius with 5 minutes. Finally, the designed MFC was obtained after the channels cleaning by gas.

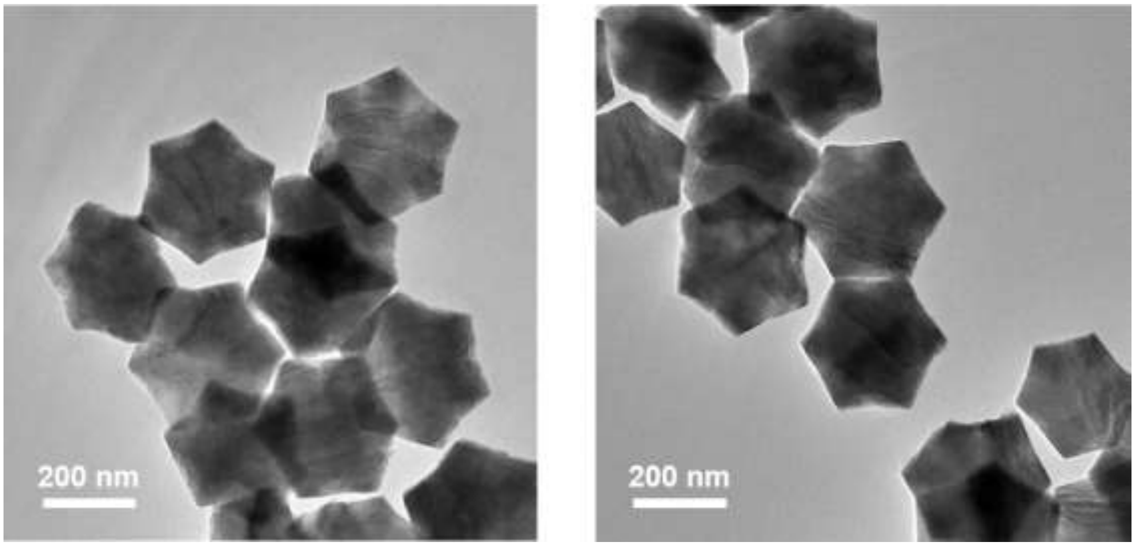

Figure S1. The TEM images of $NaYF_4$:Er^{3+}/Yb^{3+} UCNPs after acid treatment.

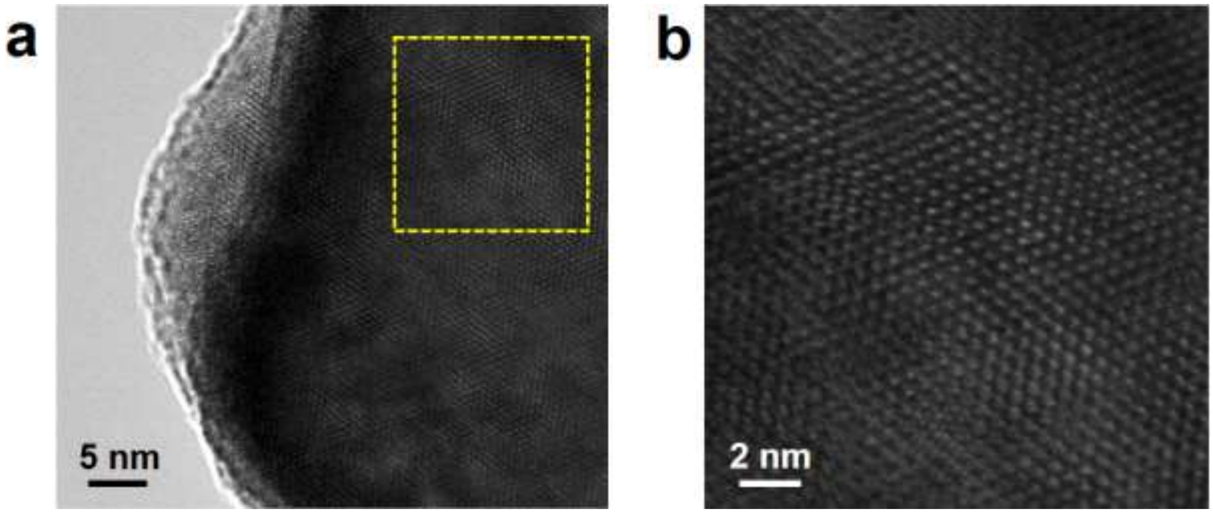

Figure S2. The HR-TEM images of prepared $NaYF_4:Er^{3+}/Yb^{3+}$ UCNPs with (a) partial region pattern and (b) its selected area expended scanning image.

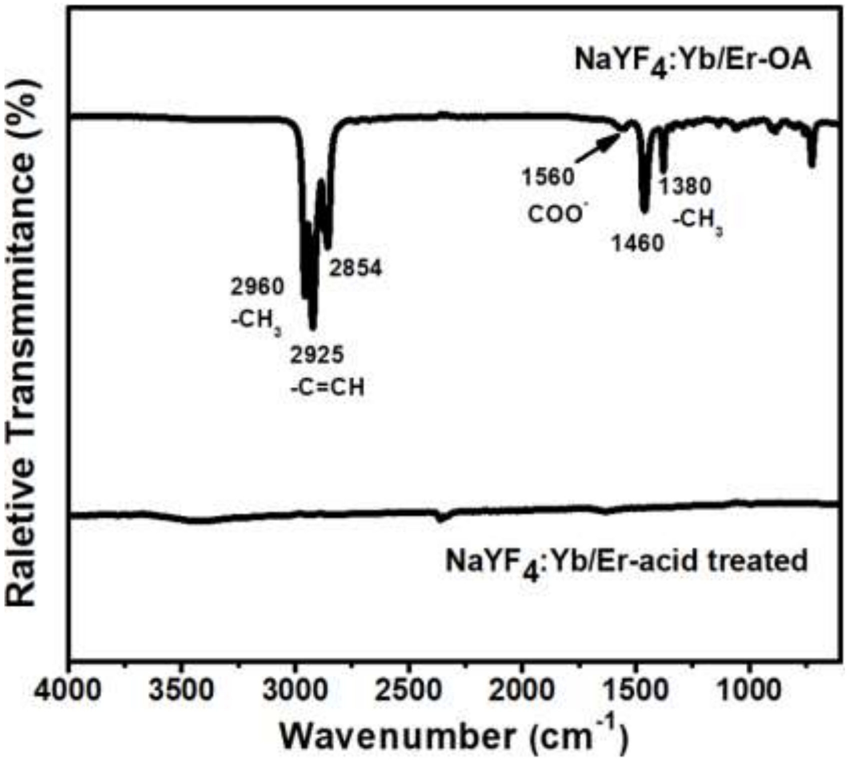

Figure S3. The FTIR spectra of $NaYF_4$:Yb/Er-OA and acid treated $NaYF_4$:Yb/Er nanoparticles.

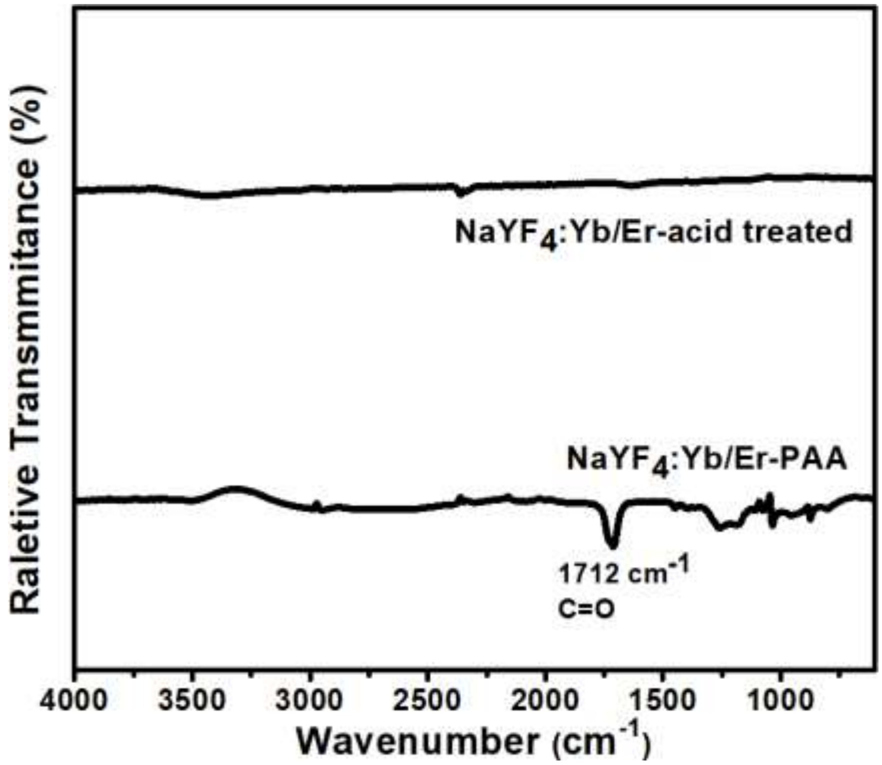

Figure S4. The FTIR spectra of acid treated $NaYF_4$:Yb/Er and modified $NaYF_4$:Yb/Er-PAA nanoparticles.

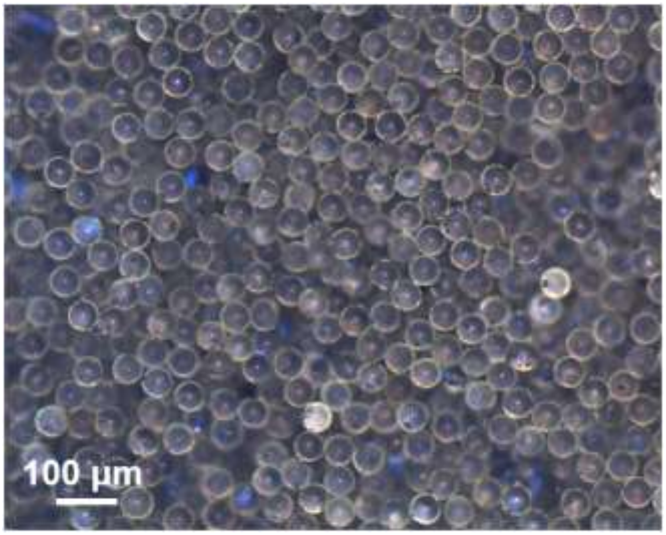

Figure S5. The acquired optical photo of PS microbeads under the reflection and bright field mode.

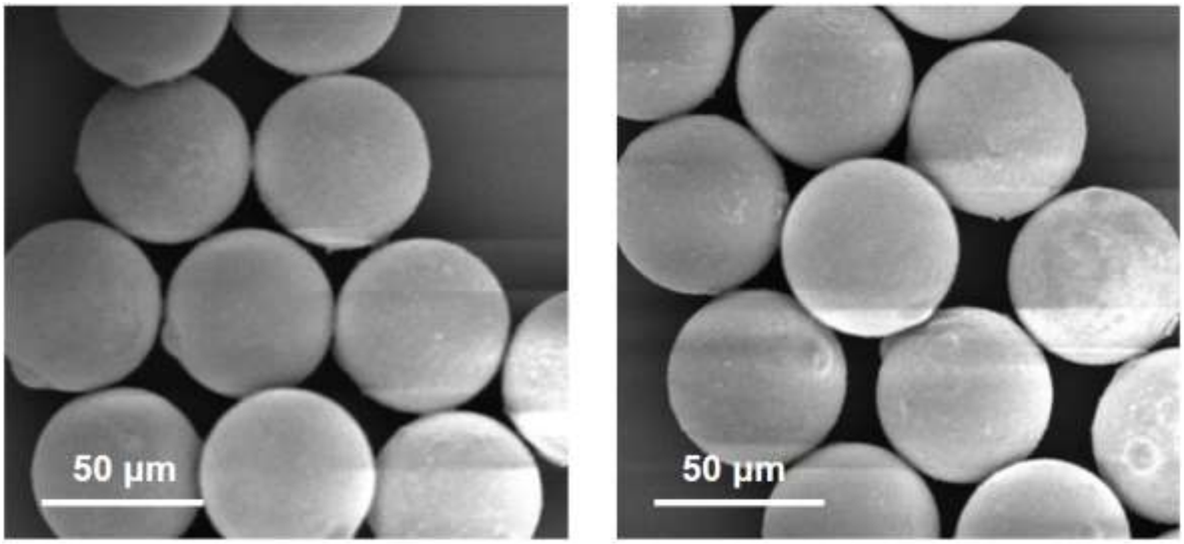

Figure S6. The SEM images of unconjugated PS microbeads under the treatment of gold coating.

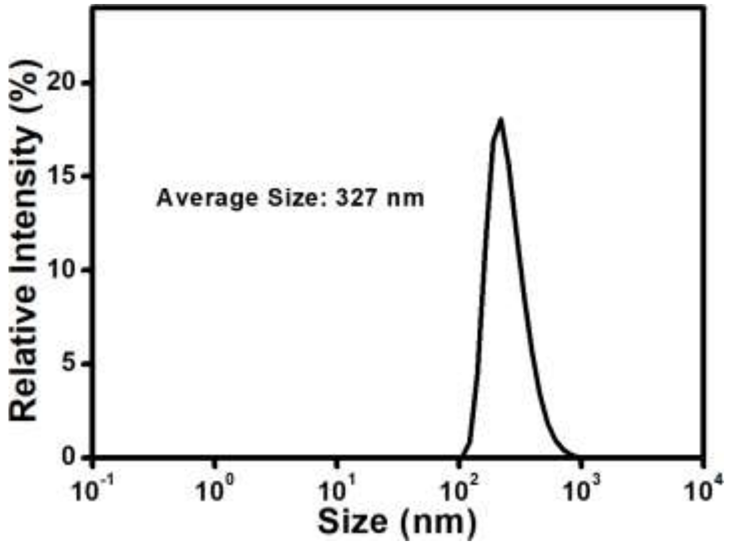

Figure S7. The DLS spectra of modified $NaYF_4$:Yb/Er-PAA and $NaYF_4$:Yb/Er-Antibody.

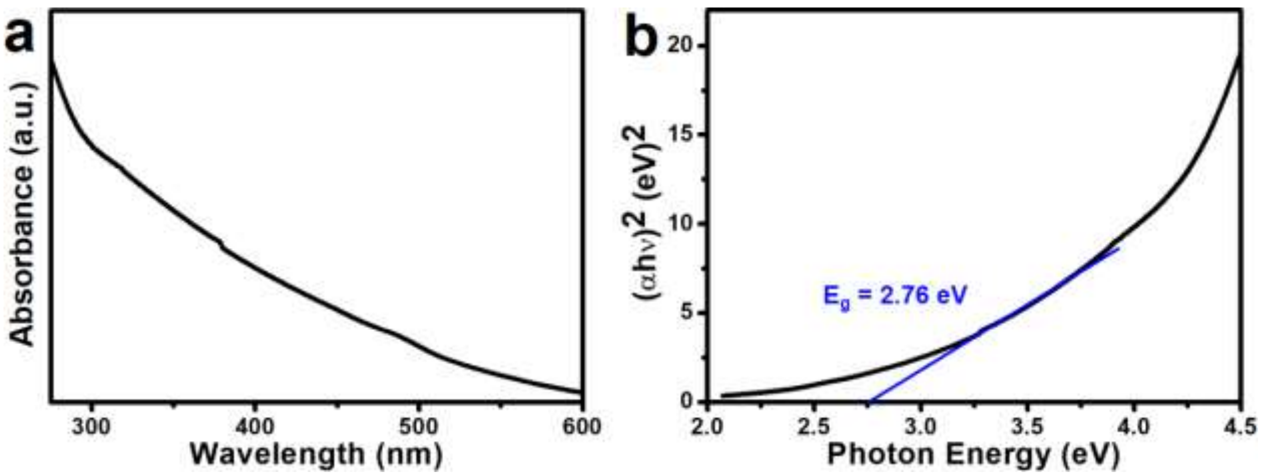

Figure S8. The absorbance spectra (a) and its Tauc-plot (b) of synthesized $NaYF_4$:Er^{3+}/Yb^{3+} UCNPs.

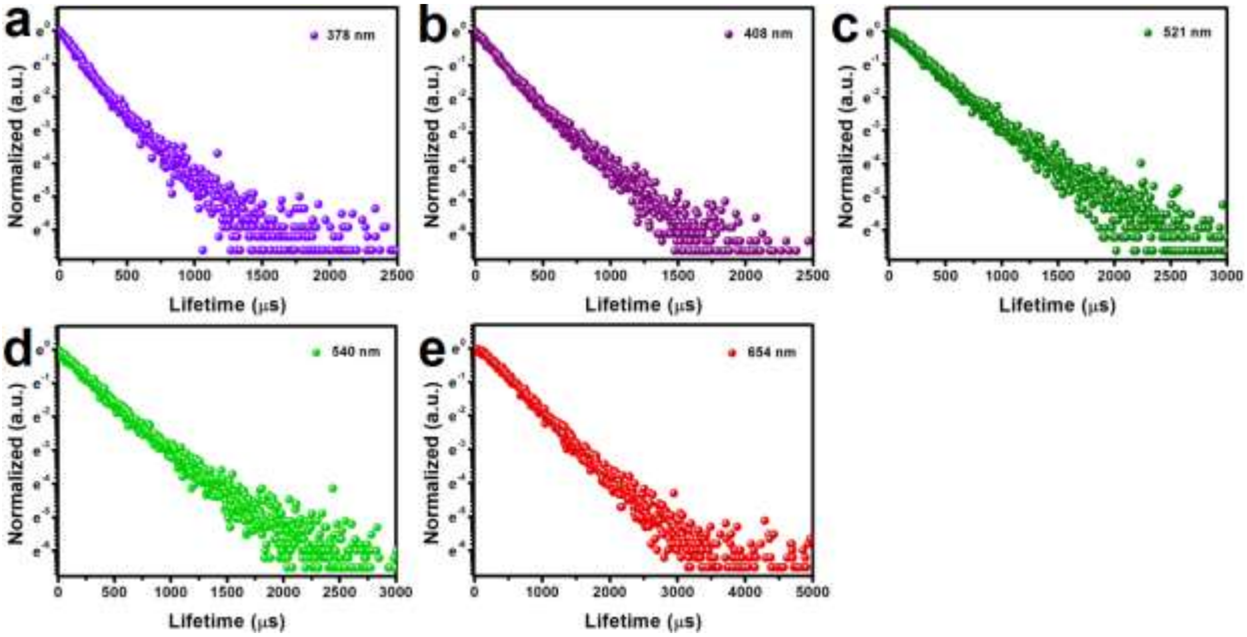

Figure S9. The photoluminescence decay time of upconversion emission at (a) 378 nm, (b) 408 nm, (c) 521 nm, (d) 540 nm, and (e) 654nm.

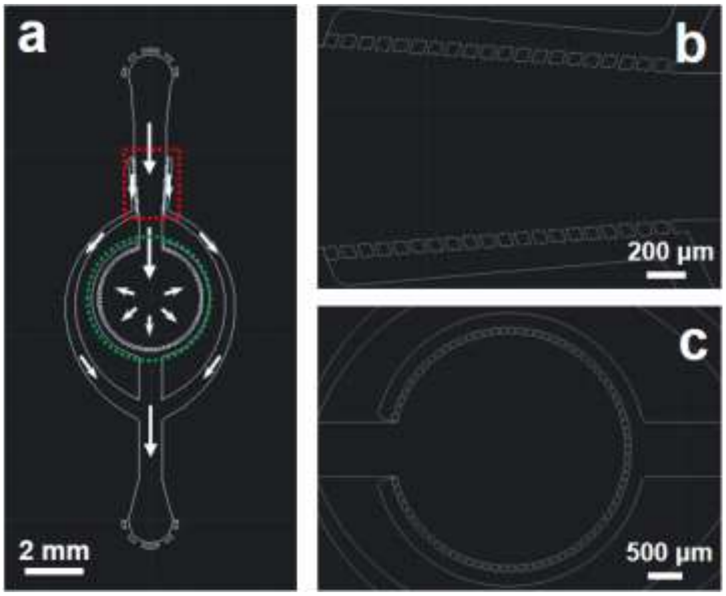

Figure S10. The AutoCAD design pattern and flow path of MFC-1 with (a) two order filtration system. The distinct channel details of red and green labelled area exhibiting at the (b) and (c), respectively.

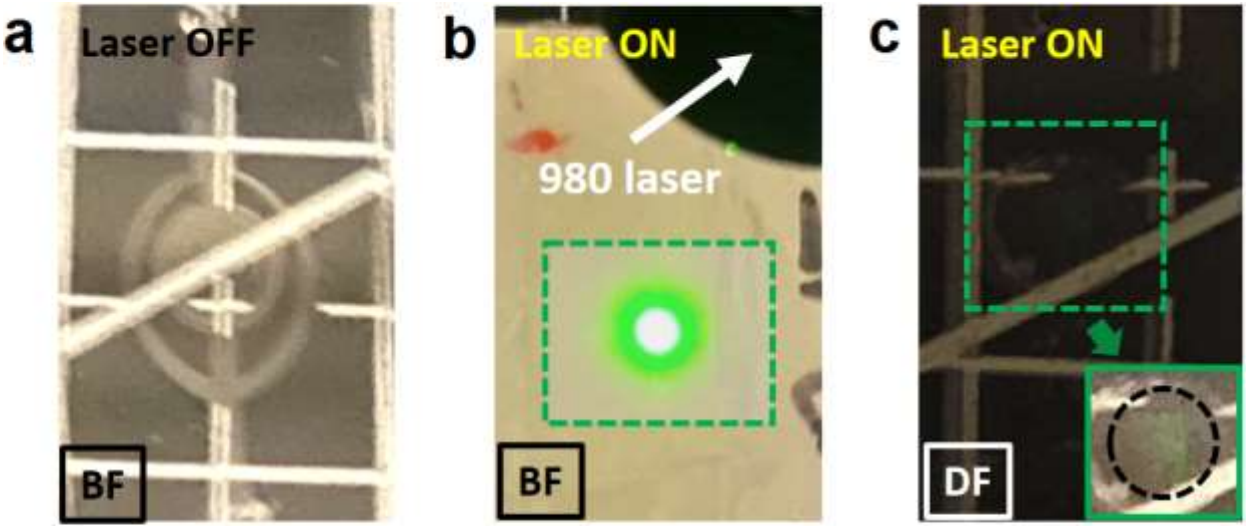

Figure S11. (a) The optical image of washed MFC-1 without laser irradiation in bright field (BF). (b) The picture of IR detection card with green emission under 980 nm laser excitation in BF. (c) The photo of washed MFC-1 under laser irradiation in dark field (DF). Bottom right inset illustrates some background signal with green emission.

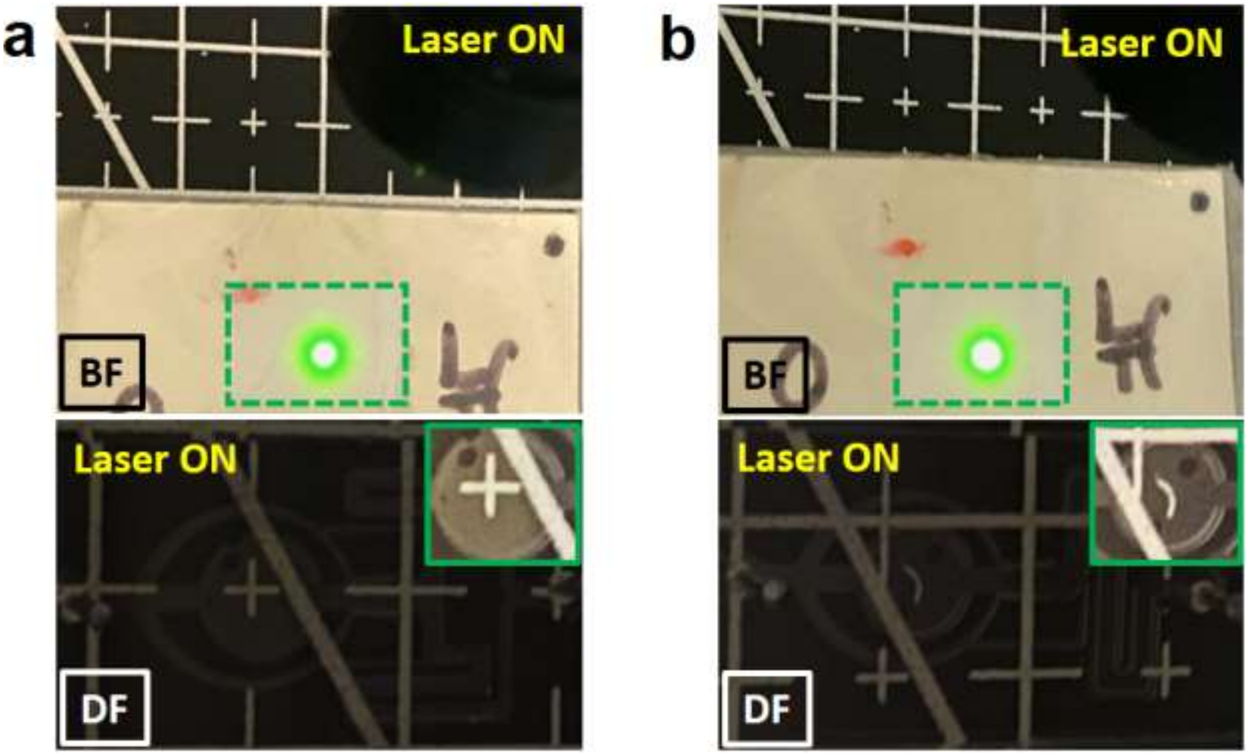

Figure S12. (a) The optical images of IR detection card (up) and washed MFC-2 (bottom) under 980 nm laser irradiation. Top right inset of bottom panel exhibits almost none background signal appearing. (b) The pictures of IR measurement card (up) and washed MFC-3 (bottom) under 980 nm laser excitation. Top right inset of bottom panel illustrates that there are hardly any background signals in central concentrating zone.

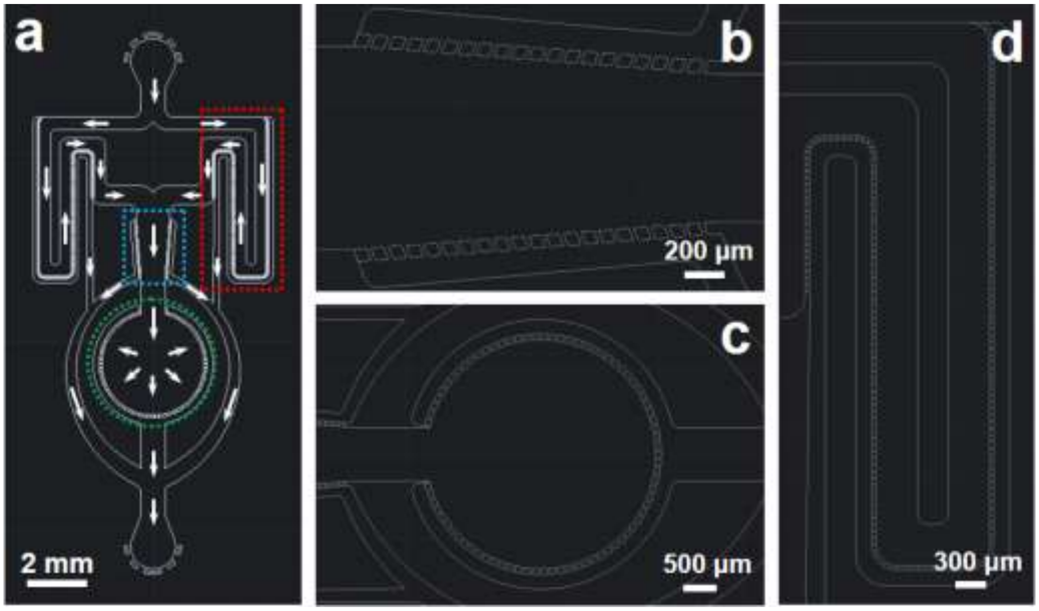

Figure S13. The AutoCAD design pattern and flow path of MFC-2 with (a) three order filtration system. The relevant detailed pattern with blue, green, and red marked area showing at (b-d), respectively.

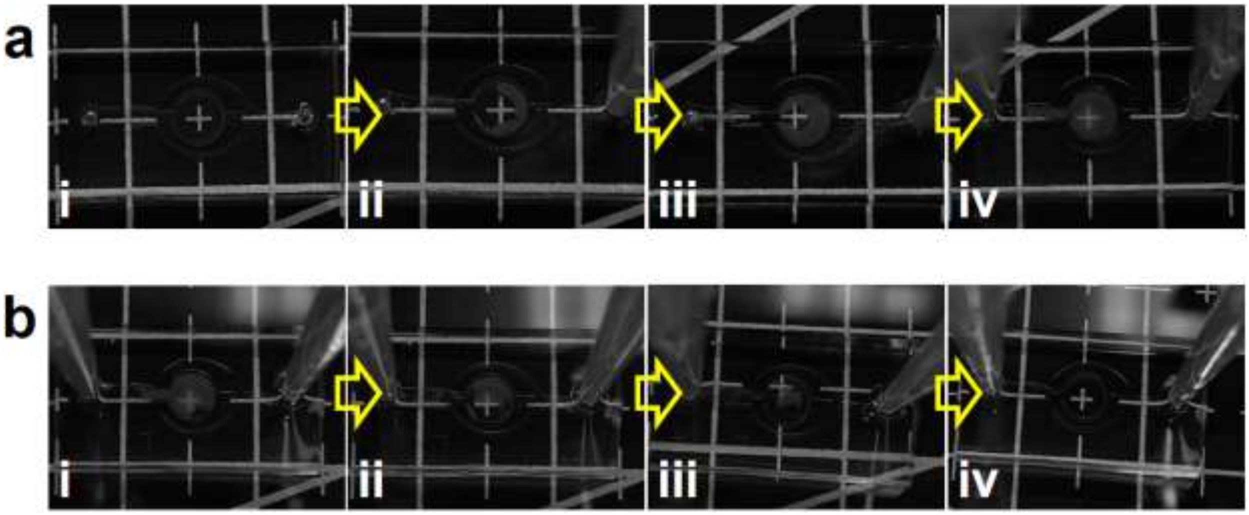

Figure S14. The optical images of separation (a) and cleaning (b) performance for MFC-1.

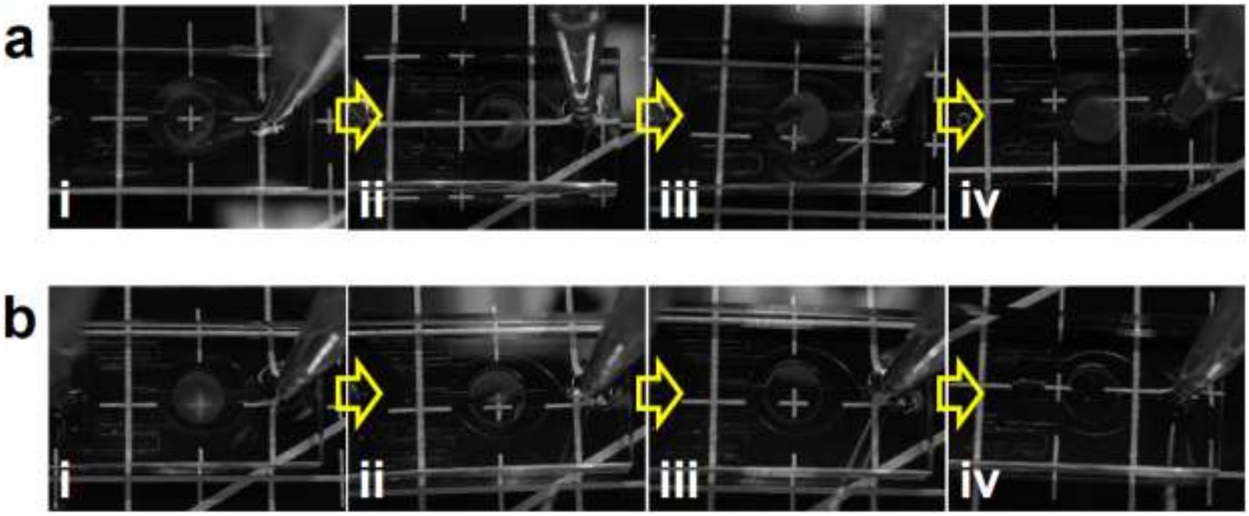

Figure S15. The photos of separation (a) and cleaning (b) property for MFC-2.

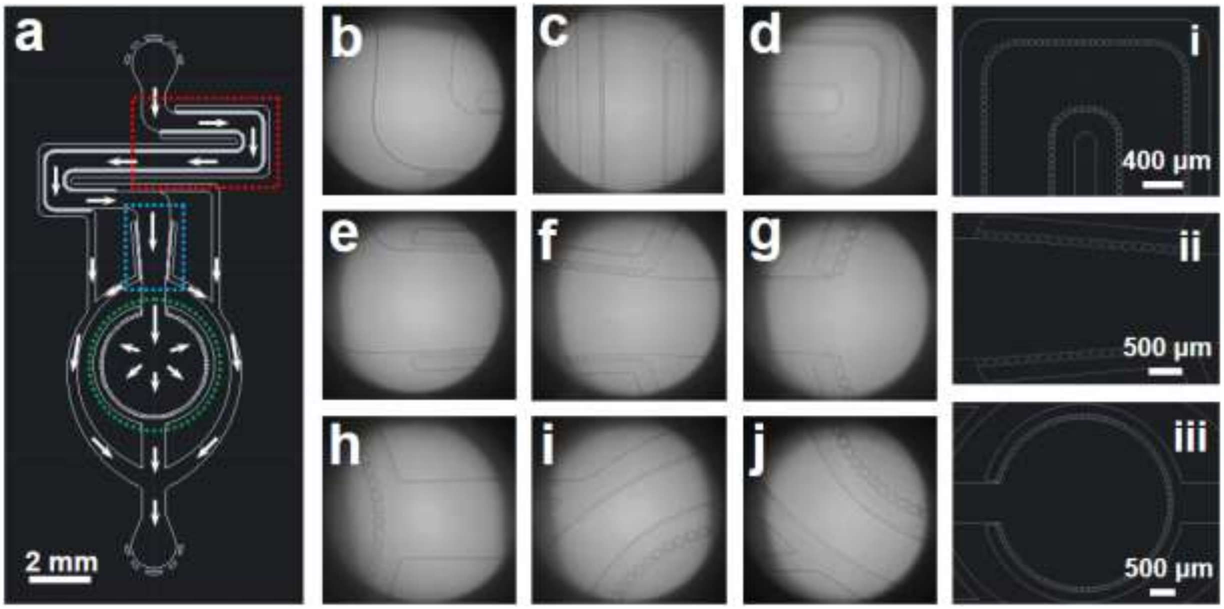

Figure S16. The AutoCAD design pattern and flow path of MFC-3 with (a) three order filtration system. The detailed pattern of three order filtration with red, blue, and green marked region showing at (i-iii), respectively. The related optical photos exhibiting at (b-j), among them, (b-c) representing the first order filtration, (e-g) illustrating the second order filtration, and the (h-j) indicating the third order filtration.

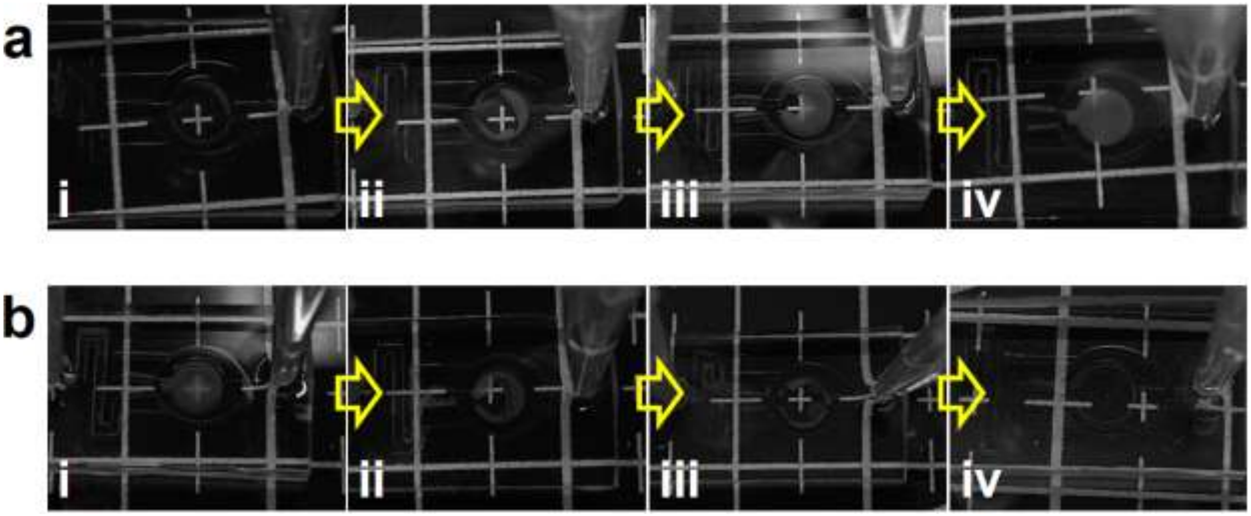

Figure S17. The pictures of separation (a) and cleaning (b) performance for MFC-1.

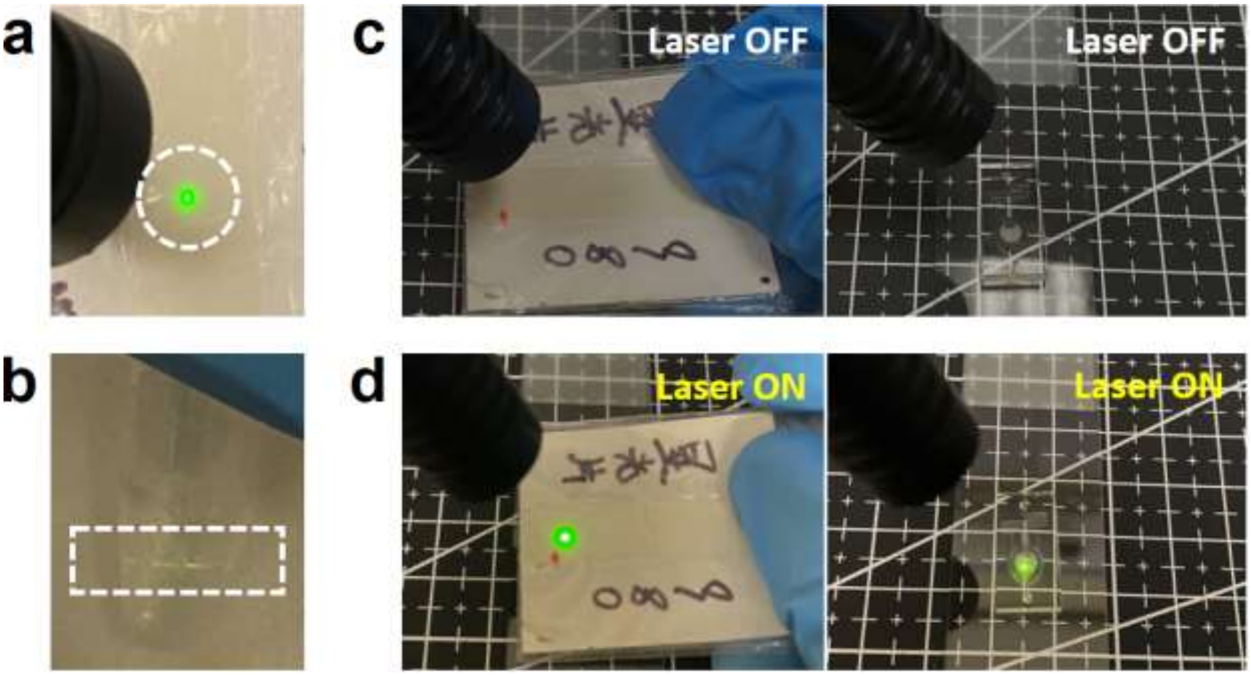

Figure S18. The related concentrating performance of microfluidic chips. (a) Picture of IR detection card with green emission. (b) Related photo of conjugated PS microbeads dispersing in liquid condition with little green emission. (c) Optical images of IR detection card (left) and concentrated conjugated PS microbeads in central zone of MFC (right) without 980 nm laser excitation. (d) Optical images of IR detection card (left) and concentrated conjugated PS microbeads in MFC (right) under 980 nm laser irradiation with green emission.

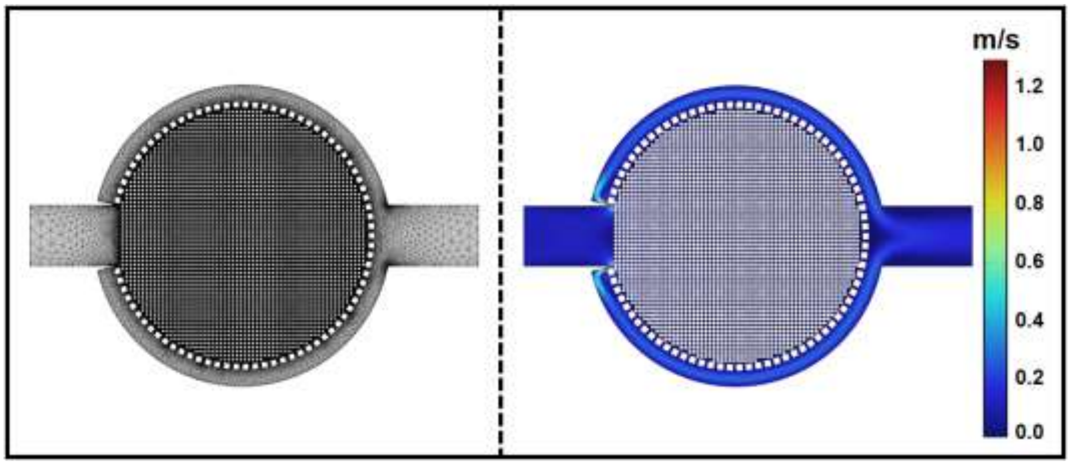

Figure S19. The relevant COMSOL simulation for designed MFC with PS microbeads.

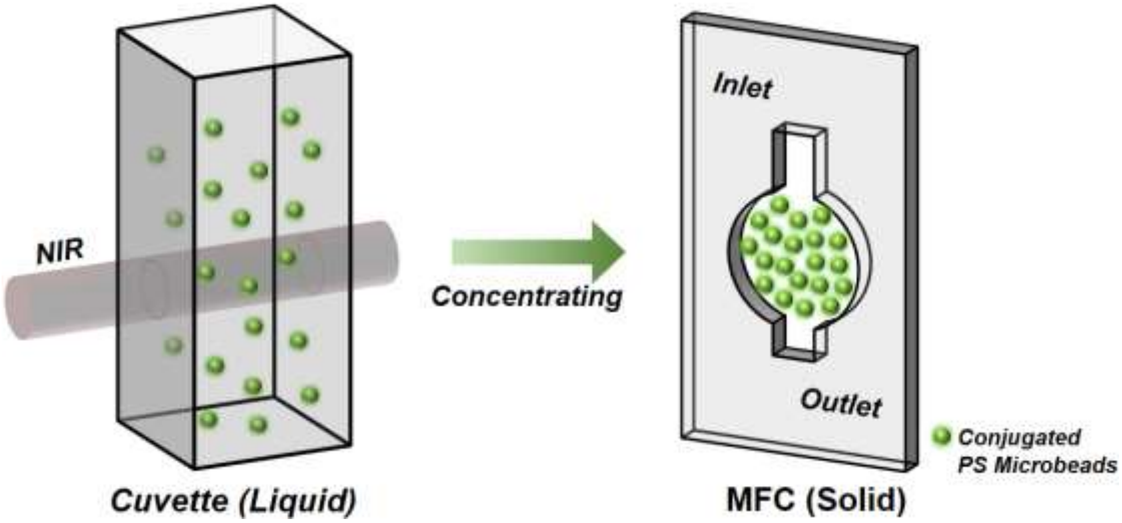

Figure S20. Schematic diagram of the MFC luminescence concentrating performance from the cuvette (liquid) to MFC (solid) mode.

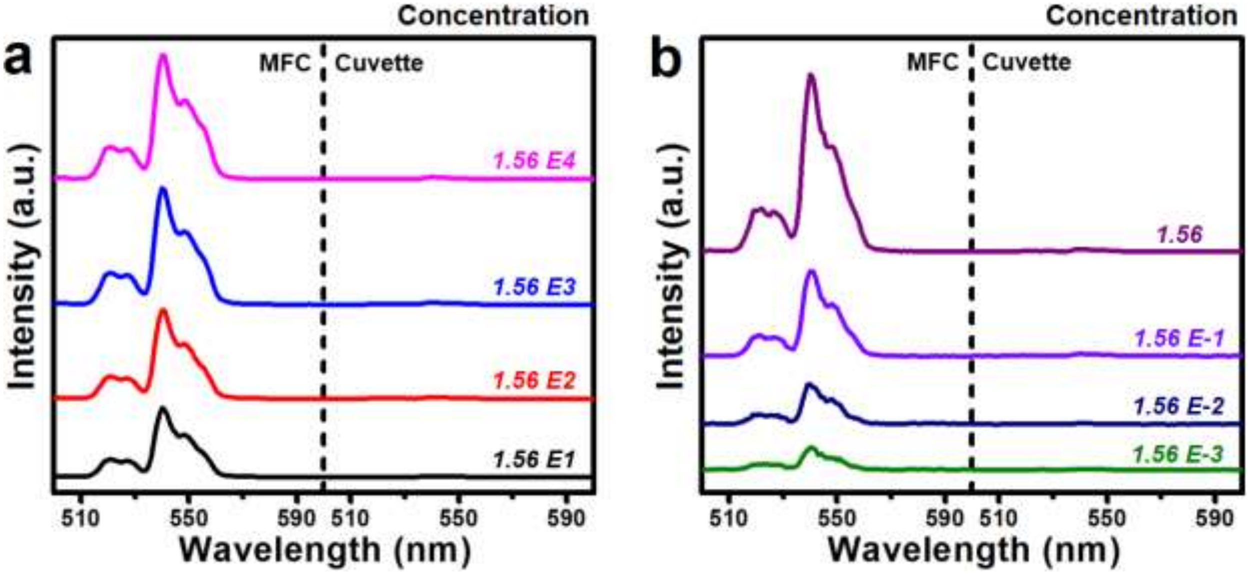

Figure S21. The compared emission spectra of MFC and cuvette mode with different virus concentration (ng/ml).

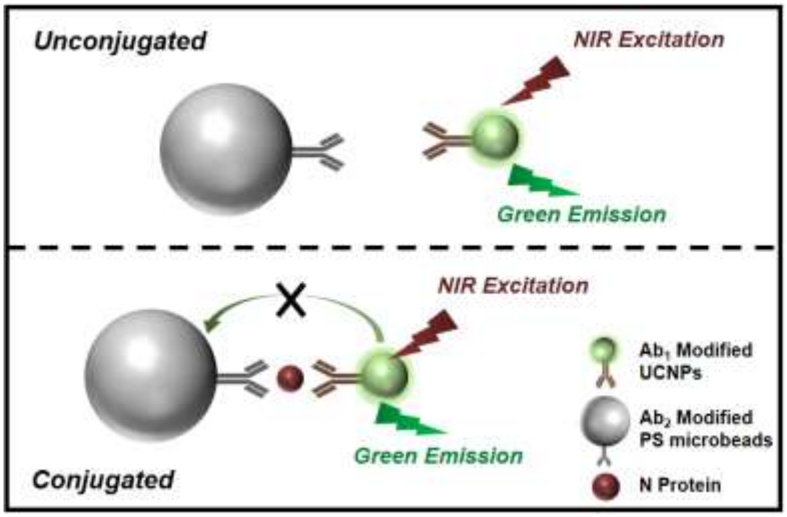

Figure S22. The schematic diagram of probable energy transfer mechanism between the unconjugated/conjugated PS microbeads and modified UCNPs.

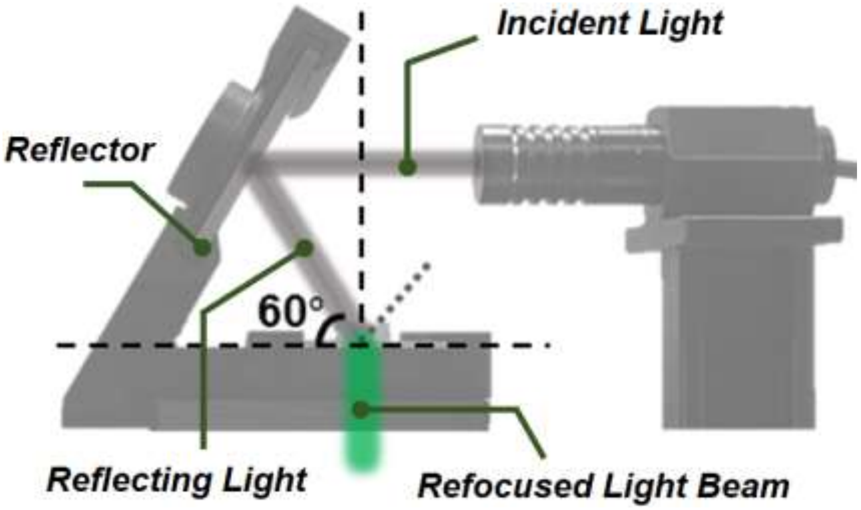

Figure S23. The designed reflected light path involving incident NIR light, reflector, reflecting light, and refocused light beam.

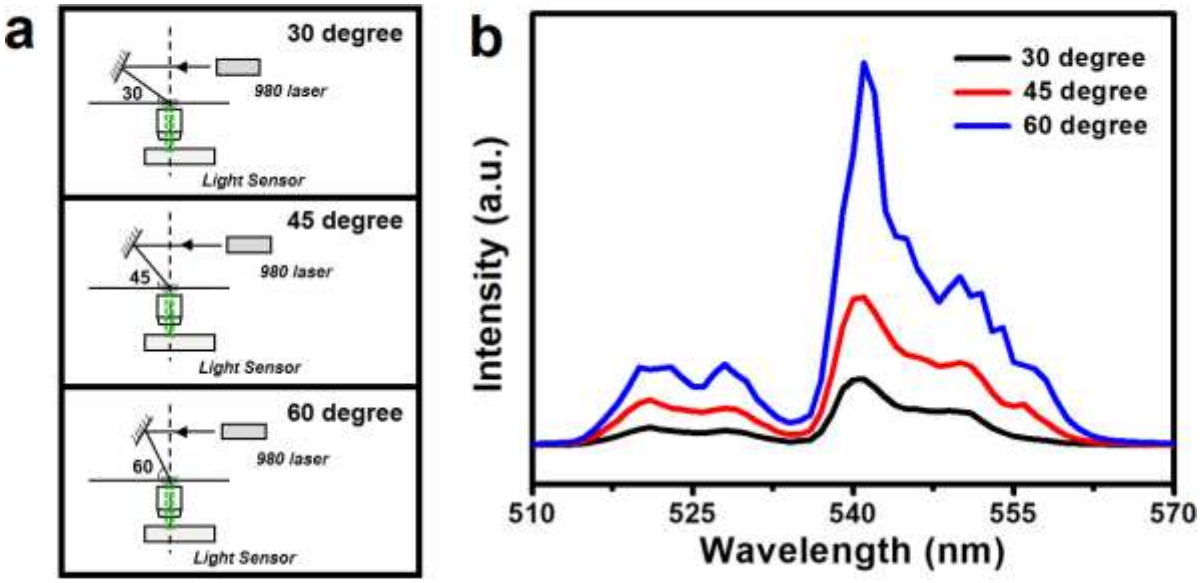

Figure S24. The schematic diagrams (a) of different light path with 30, 45, and 60 degrees. (b) The emission spectra of variant light path angles with 30, 45, and 60 degrees.

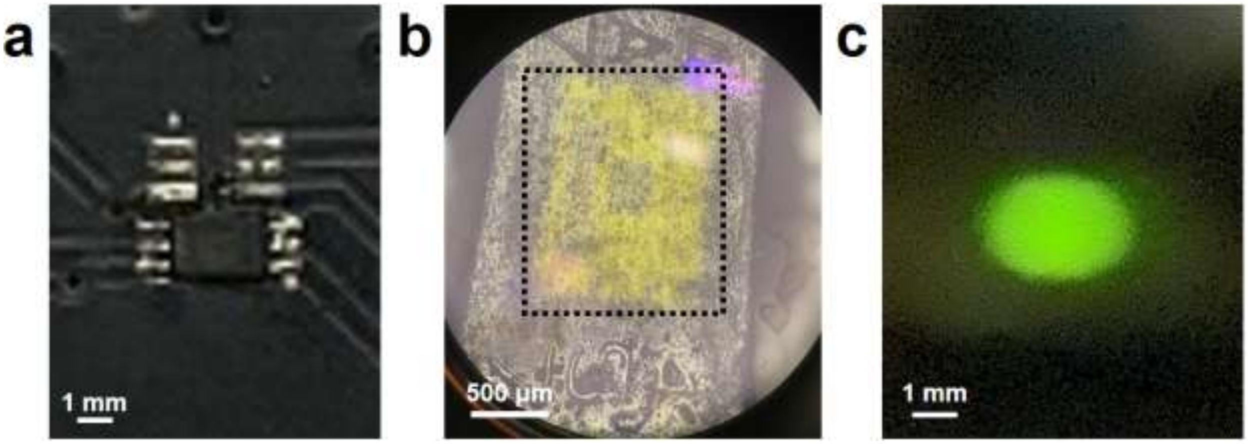

Figure S25. The integrated light sensor photos with (a) optical image of light sensor integrated with MCU. (b) The microscope photo of light sensor unit. (c) The optical spot of refocused emission light beam under the conjugated with N protein.

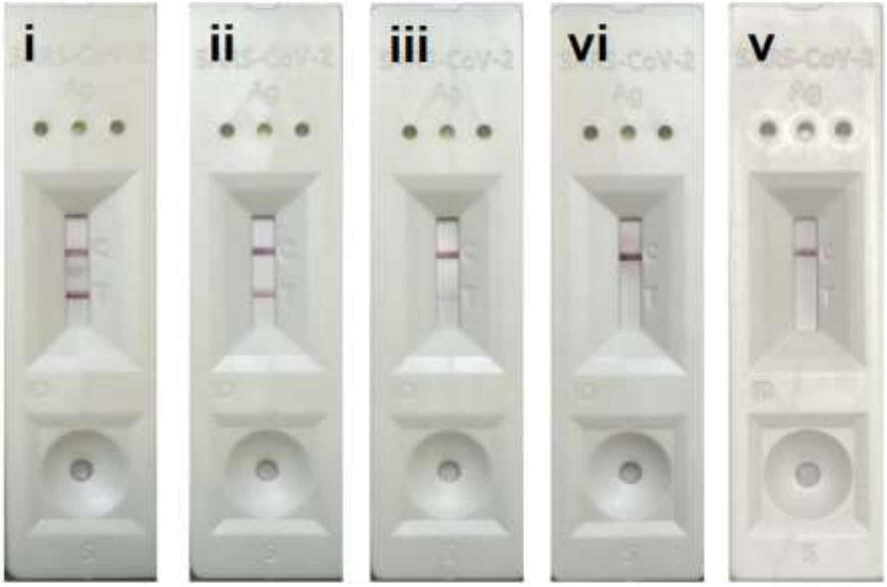

Figure S26. The optical images of commercial LFA- rapid test strips for N protein detection with different concentration.

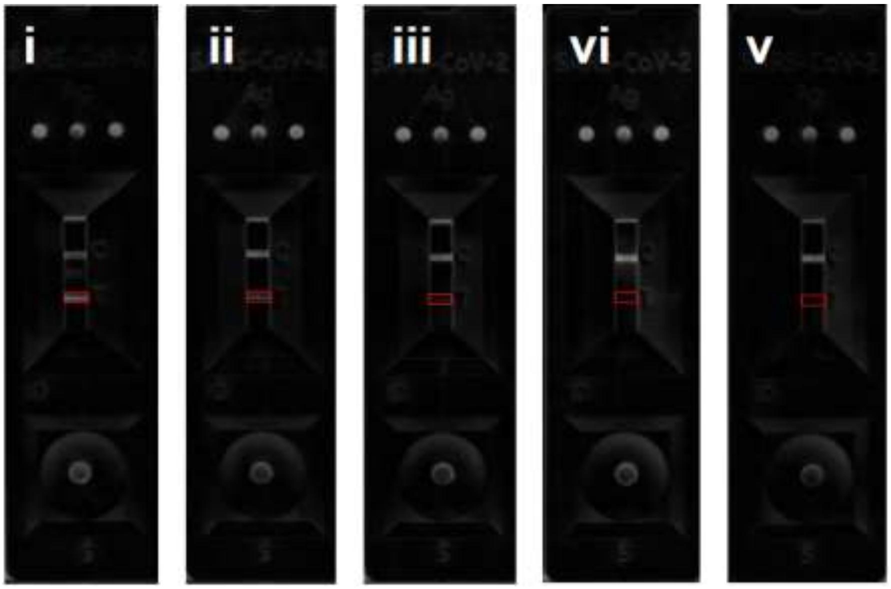

Figure S27. The converted grayscale images of commercial LFA-1 rapid test strips for N protein detection in various concentration.

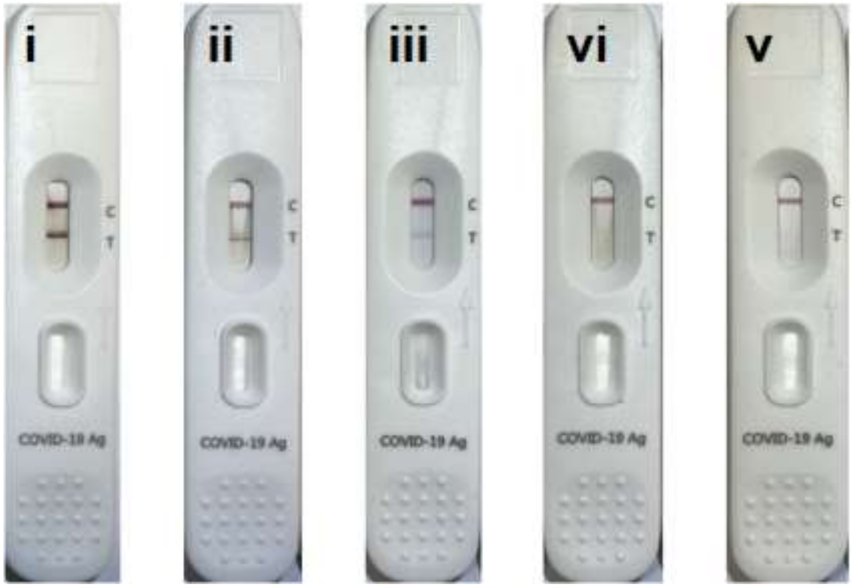

Figure S28. The optical photos of commercial LFA-2 rapid test strips for various concentration N protein detection.

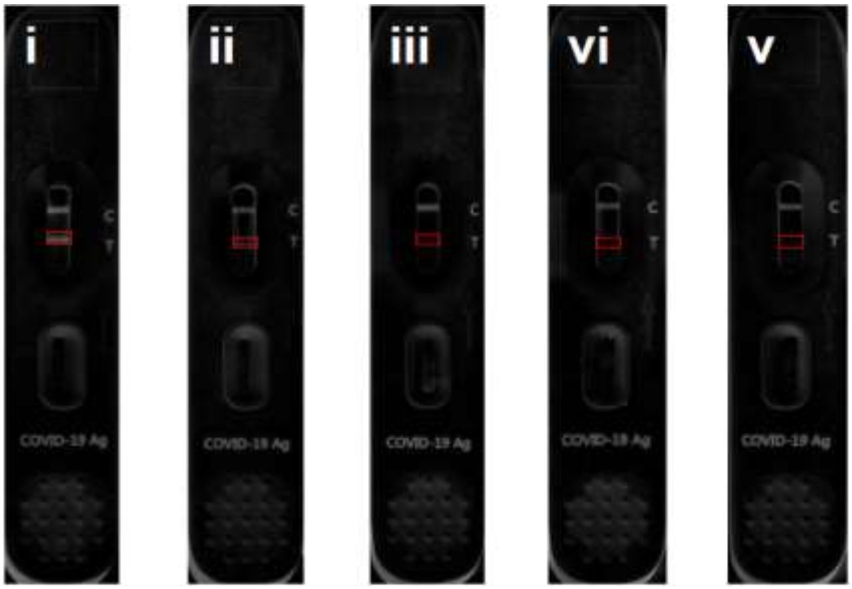

Figure S29. The related converted grayscale pictures of commercial LFA-2 rapid test strips for virus detection in different N protein concentration.

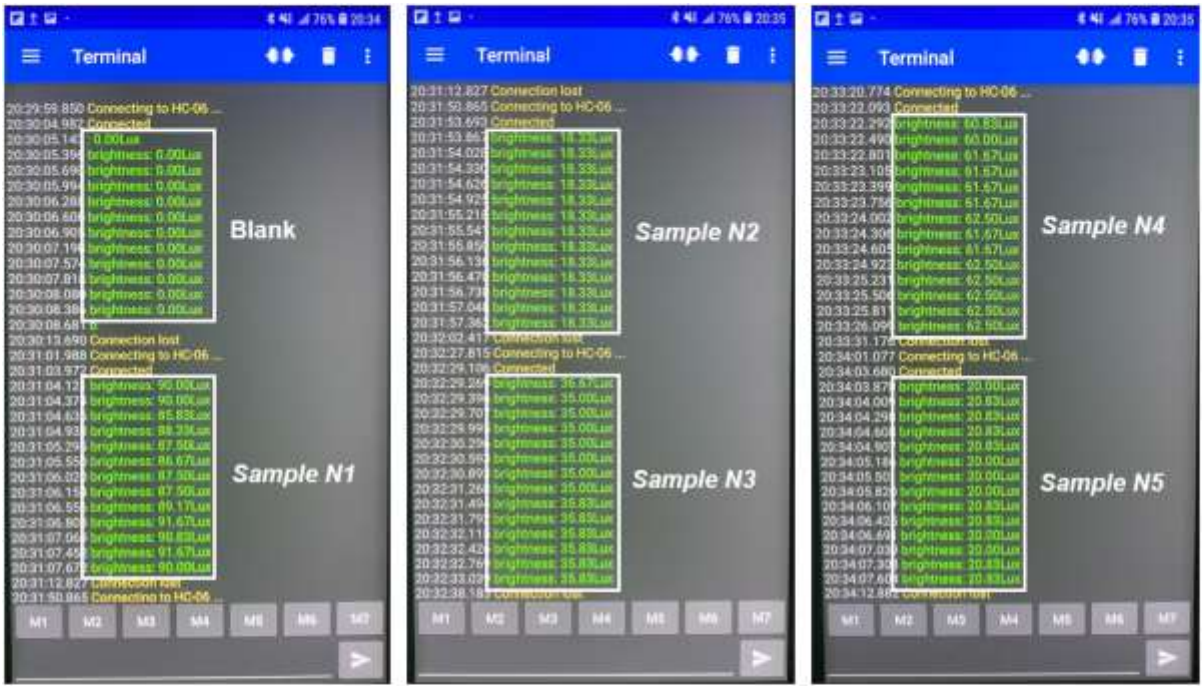

Figure S30. The mobile phone screen photos of portable device readout values for one blank test and five negative samples detection.

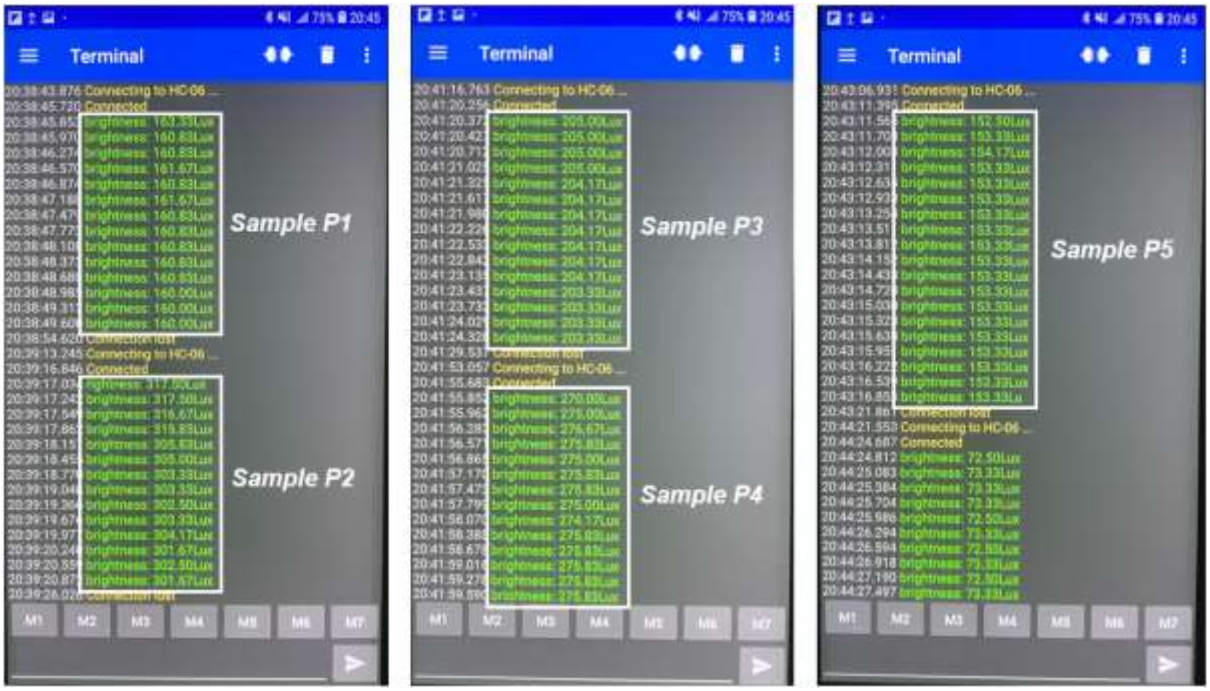

Figure S31. The optical images of portable device readout values in mobile phone for five positive samples detection.

Reference

[1] X. Zhu, J. Zhang, J. Liu, Y. Zhang, *Adv. Sci.* **2019**, *6*, 1901358.

[2] B. Amouroux, C. Roux, J.-D. Marty, M. Pasturel, A. Bouchet, M. Sliwa, O. Leroux, F. Gauffre, C. Coudret, *Inorg. Chem.* **2019**, *58*, 5082.

[3] S. Gao, J. M. Guisán, J. Rocha-Martin, *Anal. Chim. Acta* **2022**, *1189*, 338907.

[4] P. Balyan, D. Saini, S. Das, D. Kumar, A. Agarwal, *Biomicrofluidics* **2020**, *14*, DOI 10.1063/1.5143656.

[5] M. Yamada, W. Seko, T. Yanai, K. Ninomiya, M. Seki, *Lab Chip* **2017**, *17*, 304.

[6] X. Chen, D. F. Cui, C. C. Liu, H. Li, *Sensors Actuators B Chem.* **2008**, *130*, 216.
